# Initial Characterization of the Pf-Int Recombinase from the Malaria Parasite *Plasmodium falciparum*

**DOI:** 10.1371/journal.pone.0046507

**Published:** 2012-10-08

**Authors:** Mehdi Ghorbal, Christine Scheidig-Benatar, Salma Bouizem, Christophe Thomas, Genevieve Paisley, Claire Faltermeier, Melanie Liu, Artur Scherf, Jose-Juan Lopez-Rubio, Deshmukh N. Gopaul

**Affiliations:** 1 Unité Plasticité du Génome Bactérien, CNRS, UMR 3525, Institut Pasteur, Paris, France; 2 Unité Biologie des Interactions Hôte-Parasite, CNRS, URA 2581, Institut Pasteur, Paris, France; Bernhard Nocht Institute for Tropical Medicine, Germany

## Abstract

**Background:**

Genetic variation is an essential means of evolution and adaptation in many organisms in response to environmental change. Certain DNA alterations can be carried out by site-specific recombinases (SSRs) that fall into two families: the serine and the tyrosine recombinases. SSRs are seldom found in eukaryotes. A gene homologous to a tyrosine site-specific recombinase has been identified in the genome of *Plasmodium falciparum*. The sequence is highly conserved among five other members of Plasmodia.

**Methodology/Principal Findings:**

The predicted open reading frame encodes for a ∼57 kDa protein containing a C-terminal domain including the putative tyrosine recombinase conserved active site residues R-H-R-(H/W)-Y. The N-terminus has the typical alpha-helical bundle and potentially a mixed alpha-beta domain resembling that of λ-Int. Pf-Int mRNA is expressed differentially during the *P. falciparum* erythrocytic life stages, peaking in the schizont stage. Recombinant Pf-Int and affinity chromatography of DNA from genomic or synthetic origin were used to identify potential DNA targets after sequencing or micro-array hybridization. Interestingly, the sequences captured also included highly variable subtelomeric genes such as *var*, *rif*, and *stevor* sequences. Electrophoretic mobility shift assays with DNA were carried out to verify Pf-Int/DNA binding. Finally, Pf-Int knock-out parasites were created in order to investigate the biological role of Pf-Int.

**Conclusions/Significance:**

Our data identify for the first time a malaria parasite gene with structural and functional features of recombinases. Pf-Int may bind to and alter DNA, either in a sequence specific or in a non-specific fashion, and may contribute to programmed or random DNA rearrangements. Pf-Int is the first molecular player identified with a potential role in genome plasticity in this pathogen. Finally, Pf-Int knock-out parasite is viable showing no detectable impact on blood stage development, which is compatible with such function.

## Introduction

*Plasmodium falciparum* is among the main causative agents of malaria in humans, and is responsible for over 200 million infections and 600,000 deaths annually [1]. The ability of the parasite to generate tremendous genomic plasticity is a major obstacle in disease control. The underlying mechanisms at the heart of this paradigm are not sufficiently understood. This concerns in particular the virulence gene families that encode variant proteins such as Pf-EMP1, Rifin, and Stevor at the surface of the infected red-blood cell (RBC) [2]. Approximately 60 *var*, 150 *rif* and 30 *stevor* genes are dispersed throughout the genome [2], [3]. These genes are located sub-telomerically, where they may benefit from the intrinsically variant nature of the telomeres for increasing in diversity. It has been proposed that the tethering of *var* genes to the nuclear periphery, forming foci of 4–7 chromosome ends and internal *var* may enhance recombination [4]. However, the observation that the rate of acquisition of genetic diversity in the *var* genes between lifecycles in the host is faster than expected from Mendelian transmission alone [4] suggests that other factors might be involved. The presence of a recombination machinery that specifically contributes to this phenomenon in *P. falciparum* analogous to what exists in *Borrelia* [5] or *Nesseria* [6], has not yet been identified. Although homologous recombination is predicted to be responsible for changes occurring *in trans*, intra chromosomal recombination *in cis* involving upstream regions of *var* genes has also been observed [7]. Proteins involved in homologous recombination, such as Rad51, have been proposed to be involved in antigenic variation in kinetoplastid protozoan parasites [8], but their role is still unclear [9]. The Rad51 independent recombination has also been shown to only require short DNA stretches (7–13 bp) [10]. Precise short DNA region dependent recombination is the hallmark of site-specific recombinases, which use targets with a high level of homology and with palindromic symmetry [11]. In some cases, the DNA substrates could also be in the form of refolded single stranded DNA with imperfect symmetry [12]. Depending on the organization of the recombination sites, reactions result in rearrangement of DNA segments by integration, excision, or orientation alteration [13]. Site-specific recombinases are involved in vital DNA transactions in the cell, including genome repair, replication and recombination. They can be frequently found in prokaryotes; however, little information exists on their presence or role in eukaryotes.

A search of the *Plasmodium* genome sequencing project database PlasmoDB [14], [15] has allowed us to identify a phage integrase-like domain within a 490 amino acid (aa) open reading frame at the locus Mal13P1.42, which we named Pf-Int. Sequence alignments [16] using members of the tyrosine site-specific recombinase (Y-SSR) family confirmed that Pf-Int contains the R-H-R-H/W-Y active site motif in its C-terminal region corresponding to aa 298–490. Y-SSRs use an active site tyrosine residue in order to cleave at a scissile phosphate on their target sites. The exchange of the first DNA strand of the duplex in the Y-SSR generates a Holliday junction (HJ) intermediate which is resolved into recombinant products [17]. In the case of Serine recombinases a 5′ transient covalent link is formed with both strands of the duplex undergoing nucleophilic attack in a staggered manner.

Here, we present the biochemical characterization of the eukaryotic Y-SSR Pf-Int that is expressed in the blood stages of *P. falciparum* and conserved among several members of the Plasmodia. We identified potential DNA target sites using purified recombinant Pf-Int protein to enrich for DNA-binding. We also present here, the functional characterization of a parasite line where the Pf-Int gene was knocked out.

## Results

### Identification of Pf-Int

**Figure 1:**
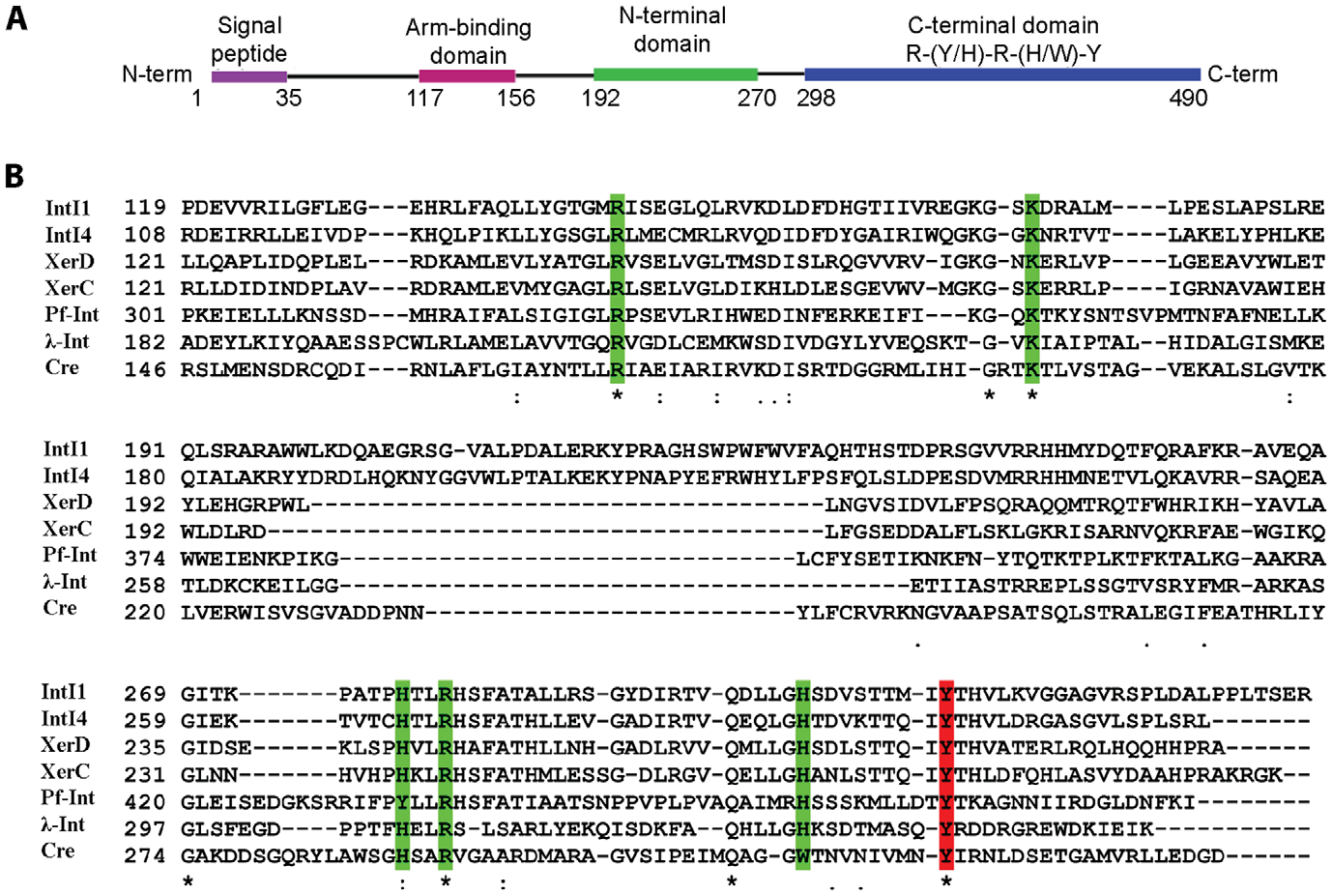
Pf-Int is a tyrosine recombinase. **A**) Schematic representation of domain organization of Pf-Int. The integrase is 490 amino acids long and is predicted to have two DNA-binding domains, the Arm binding domain (117–156 aa residues) and the core-binding domain (192–490 aa residues). The core binding domain is made of the two domains classically found in Y-SSR: the N-terminal domain (green) and the C-terminal catalytic domain (blue) that contains the catalytic residues R-K-(Y/H)-R-H/W-Y. **B**) Sequence alignment using Pf-Int and members of the tyrosine recombinase family (λ-integrase, Cre, XerD, XerC, IntI1, IntI4). Conserved catalytic residues (R-K-H-R-H/W) are depicted in green. The catalytic tyrosine is depicted in red.

PlasmoDB database mining has enabled the identification of an integrase-family coding open reading frame of 490 aa (locus Mal13P1.42). Sequence alignments with other members of the tyrosine recombinase family (e.g. λ-Integrase, bacteriophage P1 Cre, *E. coli* XerC, XerD, *Vibrio cholerae* VchInt1b (Int4), and *Pseudomonas aeruginosa* Int1) showed high homology, both in terms of conserved active site residues and polypeptide length, corresponding to the region comprised of aa ∼192–490 of Pf-Int (Figure 1A and 1B). Using both sequence alignments with the above-mentioned Y-SSR family members and 3D structure threading (Accelrys, Modeler) on λ-Integrase (PDB-id 1Z1B) as a model, the predicted tertiary structural organization is consistent with an N-terminal α-helical region from aa ∼192–270, a linker from aa ∼271–297, and a C-terminus from aa ∼298–490. This assignment is compatible with the classical Y-SSR family fold [11], [13] and also in close agreement with the prediction by PlasmoDB for the same segments. Additionally, aa ∼117–156 potentially corresponds to the “Arm-DNA” binding region of λ-Integrase [18]. The C-terminal part of the Y-SSR has the most conserved fold bearing their active site residues (R-K-H-R-(H/W)-Y) [17]. For Pf-Int, this includes the aa set R328, K350, Y435, R438, H463 and Y473 (Figure 1B). Thus the first five residues could participate in surrounding the scissile phosphate for stabilization and protonation of the leaving 5′OH, and most importantly aa Y473 potentially serves as the attacking nucleophilic tyrosine. It can be noted that aa Y435 in Pf-Int substitutes for the first histidine of the consensus. As a possible alternative, a histidine nearby (aa 439) can potentially perform the same role. This redundancy for the first histidine 435 exists in the case of the integrases Int1, Int4, and XerC/D, however, these do not contain the initial H-to-Y substitution. In addition to the canonical residues, G326, E331, and L437, match other highly conserved positions [19].

The Pf-Int sequence has been found to be present and highly conserved among plasmodia as more genome sequences are being released. Thus, *P. berghei*, *P. chabaudi*, *P. knowlesi*, *P. yoelii*, and *P. vivax*, show 74%, 72%, 71%, 72%, and 70% identity respectively, with the most conserved being the aa 151–490 region (88%, 87%, 90%, 88%, and 90% identity respectively) (Figure S1A). Paralogues in *Toxoplasma gondii* [20], *Neospora caninum* (GenBank accession numbers: XP_002365137 and CBZ52941, respectively) and partially for *Eimeria tenella* (GeneDB accession number ETH_00001505) have also been identified (Figure S1B). Interestingly, we observe that the substitution H435Y is conserved throughout the other members of the Plasmodia and Apicomplexa (Figure S1A and B). The full length recombinase sequence includes an approximately 100 amino acid stretch that contains a potential apicoplast targeting signal that could mediate the targeting of Pf-Int to this essential organelle, and a lysine and arginine rich patch that could direct it to the nucleus.

### Erythrocytic life-stage expression of Pf-Int

Having laid the basis of Pf-Int carrying the necessary elements to act as a recombinase from the primary structure and corroborating 3D data from λ-Integrase, we sought to determine its potential role as a novel recombinase in *P. falciparum*. Pf-Int m-RNA expression levels at different stages during the erythrocytic cycle (rings, trophozoite and schizonts) were monitored by qRT-PCR from synchronized parasite cultures using the *P. falciparum seryl-tRNA synthetase* gene (*PF07_0073*) as internal control. Differential expression of Pf-Int mRNA levels was observed, (Figure S2A). A five-fold increase during the trophozoite stage and ultimately ten-fold in the schizont stage were observed compared to ring stage. We attempted further analysis by following Pf-Int basal protein expression from cell extracts by Western blotting. Using antibodies raised against Pf-Int-C162 (a truncated Pf-Int comprising aa 162–490), we were not able to clearly assess the expression of the protein by the parasite. Using cell extracts of *P. falciparum* asexual stages, *P. falciparum* gametocytes, *P. berghei* asexual stages and *P. berghei* sporozoites (Figure S2B) we observed a band whose size could correspond to the predicted molecular weight (52 kDa) of the integrase in the *P. berghei* sporozoites (Figure S2B, lane 6). In addition, the episomal expression of a Flag-tagged Pf-Int from an exogenous promoter could not be detected (data not shown). Thus, the recombinase is probably expressed either at a low level, or at a different stage of the life cycle.

### Knock-out mutagenesis of Pf-Int in *P. falciparum*

**Figure 2:**
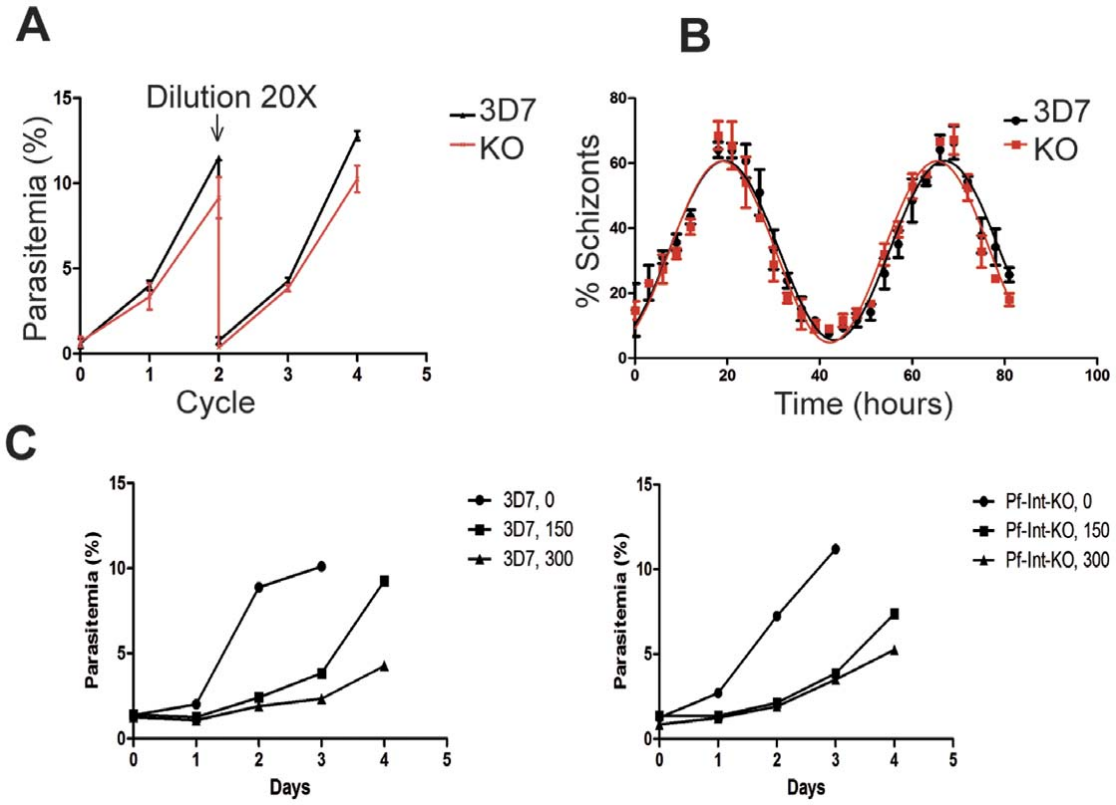
Pf-Int is non-essential for the intra-erythrocytic parasite growth. **A**) The effect of the gene disruption was examined by comparison of synchronized cultures of WT (black line) and KO (red line) parasites for four cycles. The cultures were diluted 20 times at the end of the second cycle. Values are the mean of two independent experiments whose standard deviation is shown by the bars. **B**) Synchronous cultures of the WT (black circles) and KO (red squares) parasites were grown for 80 hours and sampled every 3 h. The parasite stages were analyzed by flow cytometry and the percentage of parasites at the schizont stage was plotted against time. Values are the mean of two independent experiments whose standard deviation is shown by the bars. The data were fitted to an exponential sine wave equation using Prism program, and the fitting curves are shown. Black line corresponds to the WT cultures, and the red line to the KO cultures. **C**) **Response to UV-induced DNA damage in 3D7 and Pf-Int-KO parasites.** The effect of the gene disruption was examined by comparison of WT 3D7 (Left panel) and Pf-Int-KO (Right panel) parasites after UV irradiation at different doses (0, 150 and 300×100 µJ/cm^2^). Synchronous cultures were grown for 4 days after irradiation and sampled every day. The parasitemia was determined by flow cytometry. One representative experiment of two is shown.

To investigate the biological role of Pf-Int, we established a parasite line where Pf-Int gene was disrupted by double crossover recombination. The pCC1-Pf-Int vector contains the hdhfr gene flanked by 5′ and 3′ segments (box 1 and box 2) of the Pf-Int gene (Figure S3A). 3D7 parasites were transfected with the pCC1-Pf-Int construct and selected on WR99210 and ganciclovir to generate an insertional disruption mutant, the Pf-Int-KO. After drug selection, the Pf-Int-KO was cloned by limiting dilution and genetically characterized. Clones were screened by PCR analysis for the disruption of the Pf-Int gene as well as for the absence of the contaminating wild type gene (Figure S3B). Transcription of the gene was analyzed by Northern blot using Pf-Int specific probes. No transcript was detected in the Pf-Int-KO line (Figure S3C). The KO parasites were viable and there was no obvious difference in the parasite cell morphology or in parasite growth compared to the 3D7 parasites. Indeed, Pf-Int-KO parasites showed a cell cycle time and a multiplication rate comparable to those of the 3D7 line (Figure 2A, 2B). This suggests that the Pf-Int protein is not essential for intraerythrocytic parasite development *in vitro*.

Additionally, in order to investigate if Pf-Int plays an active role in the response to DNA damage, we performed a dose/response study to UV-induced DNA breaks. After UV irradiation, the cultures were followed by determination of parasitemia for 4 days. 3D7 (Figure 2C left panel) and Pf-Int-KO (Figure 2C, right panel) parasites showed the same overall profiles with a recovery at day 4 post-treatment.

### Cloning and expression of Pf-Int in *E.coli*

Sub-regions corresponding to aa 162 to 490, and aa 192 to 490 were used as constructs for *Escherichia coli* expression. Pf-Int-C162 designed initially with the classic tyrosine recombinase fold in mind stopped short of the lysine residue stretch (aa 152–157) and has a theoretical mass of 38,352 Da. Alignment with several tyrosine recombinase family members, including λ-Integrase, led to the construction of Pf-Int-C192. Both constructs included the α-helical bundle of the N-terminus and the C-terminal catalytic domain (aa 298–490). The constructs contained either N- or C-terminal 6-histidine tags for protein purification purposes. Expression and purification of Pf-Int-C162 with a C-terminal His-tag produced a ∼39 kDa band as determined by SDS-PAGE, as expected. The N-terminal His-tag version, after cleavage of the tag and purification to homogeneity (>95% purity), gave a ∼38 kDa band (data not shown). Analysis by MALDI-TOF (Matrix Assisted Laser Desorption-Ionization, Time of Flight) mass spectrometry yielded a mass of 38,568 atomic mass units showing that there were no exposed regions susceptible to proteases or post-translational modifications. The N-terminal His-tagged Pf-Int-C192 gave a ∼35 kDa (expected mass 35,022 Da) product by SDS-PAGE after purification and tag cleavage (data not shown). Both forms of the protein Pf-Int-C162 and Pf-Int-C192 are soluble after purification by FPLC.

### Biophysical characterization of Pf-Int

**Figure 3:**
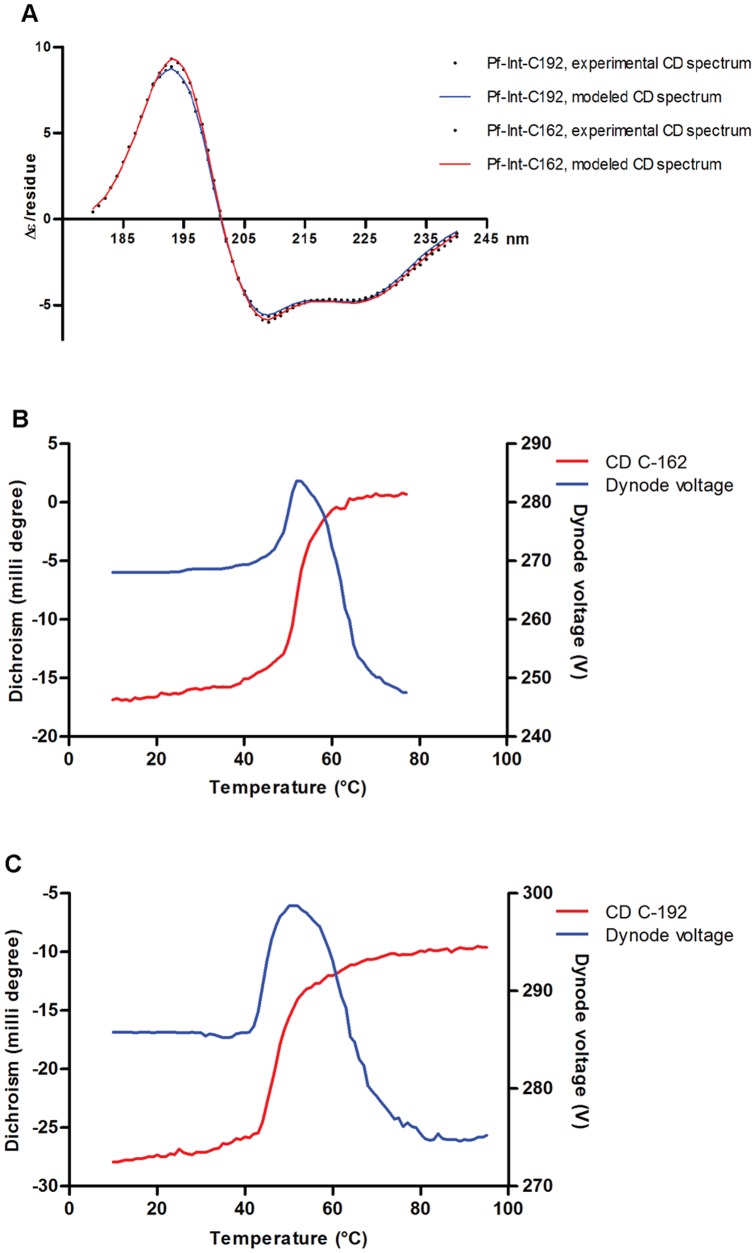
Circular dichroism analysis of the purified recombinant proteins Pf-Int-C162 and Pf-Int-C192. **A**) CD analysis in far UV. Black dots: experimental CD data. Blue line: modeled CD spectra of Pf-Int-C192. Red line: modeled CD spectra of Pf-Int-C162. The CD spectral analysis showed for both analyzed forms a high degree of α-helical organization and a low percentage of β-sheet (54/60% α-helical and 6/3% β-sheet for Pf-Int-C162/C192). **B, C**) Stability analysis by thermal denaturation coupled to CD for **B**) Pf-Int-C162 and **C**) Pf-Int-C192. Thermal CD transition curves (red lines) for Pf-Int-C162 and Pf-Int-C192 showed a beginning of stability change at approximately 50°C and 40°C, with a completion at 60°C and 50°C. The increase of the dynode signal (blue lines) indicates that the proteins are precipitating at the transition and thus the melting temperatures cannot be precisely determined.

The secondary structure contents of the purified recombinant proteins were determined by circular dichroism (CD) experiments. The CD spectral analysis of the Pf-Int constructs showed a high degree of α-helical organization and a low percentage of β-sheet (Figure 3A). Thus for Pf-Int-C(162/C192) we observed (54/60)% α-helical, (6/3)% β-sheet, (12/10)% turns, with the remainder composed of un-structured regions. These results, when compared to other members of the Y-SSR family, and especially those whose 3D structures have been solved (e.g. XerD: PDB Id-1A0P, λ-integrase: PDB Id-1Z1B, Cre: PDB Id-1CRX and IntI4: PDB Id 2A3V), showed similar overall secondary structure content. The tyrosine recombinases contain two domains (N-terminal α-helix bundle, C-terminal mixed α/β) that wrap around the core DNA sites. The two domains are connected by a linker region, which bestows the flexibility required to form a C-shaped clamp configuration on duplex DNA [21].

Both Pf-Int-C162 and Pf-Int-C192 showed typical native state behavior in their thermal CD transition curves (Figures 3B and C). We observed a beginning of stability change at approximately 50°C and 40°C, with a completion at 60°C and 50°C, which correspond to an enthalpy (ΔH) of denaturation in the range of 150 KJ/mol and 91 KJ/mol, respectively. The formation of precipitate at the transition precluded the measurement of more precise melting temperatures (Tms). Indeed, model fitting of the temperature transition requires a two-state system with a unique state at both ends. Further analysis of Pf-Int to determine its oligomerization behavior in solution was carried out. Sedimentation equilibrium showed that at all studied concentrations, Pf-Int-C162 samples sediment as a main species containing low amounts of contaminants with higher sedimentation characteristics. A sedimentation coefficient (S_20,w_) of 2.7 S and a frictional ratio f/f0 of 1.35 were determined. These values were compatible with the expected characteristics of a monomer of Pf-Int-C162 in the solution.

### DNA target search using synthetic (SELEX) and Genomic DNA (gDNA)

**Figure 4:**
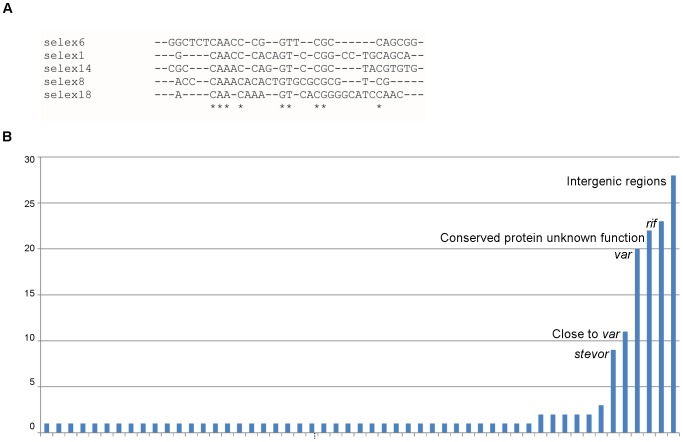
Identified DNA targets of Pf-Int. **A**) Clustal sequence alignment of the targets retained by SELEX method showed that the motif: (N)6CAANC(A/C)(N)2GT(N)3CG(N)7C(N)4 seems to be present in all of them. Conserved positions are shown by (*). **B**) Distribution of gDNA fragments retained by Pf-Int from genomic DNA capture. Y-axis: number of occurrences. X-axis: identity. Single hits are not labeled, but present in Table S2.

The identification of the DNA targets for Pf-Int was performed using two approaches. The first was to identify a minimal core half-site similar to that bound by the simplest members of the Y-SSR family. These recombinases require a short stretch of DNA (half-site) containing 13–16 bp that would be bound per monomer. The complete site is made up of a 2-fold symmetric assembly of two half-sites, flanking a central asymmetric segment of 6–8 bp [11]. The first approach therefore used an entirely unbiased DNA library composed of 25-mer variable oligonucleotides. These were screened on Pf-Int-C162 bound to Ni-NTA columns after several rounds of amplification/elution (Systematic Evolution of Ligands by Exponential enrichment, SELEX). A negative control was performed using fumarate hydratase which did not retain any DNA under our binding conditions. Five candidates were finally recovered from the sequencing of 60 colonies, which yielded the sequences shown in Figure 4A. Clustal alignments of these sequences showed conservation of their 5′ region. However, no clear motifs appeared to be present other than (N)_6_CAANC(A/C)(N)_2_GT(N)_3_CG(N)_7_C(N)_4_. Interestingly, this set contained a single hit (Selex8) that matches (13/25 bp) on a *rif* gene (3D7, chromosome10, position: 1600737 to 1600725).

As the SELEX experiment may not have benefited from the contributive effect of a second half-site adjacent to the first one, an alternative approach using 200–400 bp genomic DNA segments was adopted. Bound DNA was either ligated into plasmid vectors and submitted for sequencing, or directly hybridized onto a custom-built microarray tiled with *P. falciparum* genomic DNA [22]. Analysis of 165 colonies bearing ligated product returned 22 unique hits (Table S2A), whilst chip-hybridized sequences gave 149 hits (Table S2B). The combined results had representatives from both housekeeping and non-housekeeping genes (Figure 4B). Although a large portion of the retained sequences are from genes of the general metabolic pool, it is interesting to note that 62 hits (58 from the chip, and 4 cloned) were directly on, or in the vicinity of the *var*, *rif*, and *stevor* loci, across several chromosomes. All of the sequences that match *var*, *rif*, or *stevor* genes, or truncated forms of these were partially or totally within the extracellular domains of PfEMP1 coding exon 1 for *var*, exon 2 for *rif* or *stevor*. Clustal alignments of 300–400 bp segments comprising the hybridization site did not clearly identify a conserved segment that could correspond to a site-specific recombination site. Thus even though high NaCl (750 mM) was used in the elution steps to minimize non-specific binding, it is not clear whether the recombinase has a preference for the targets identified, or shows strong avidity for DNA due to the protein's overall positive charge (isoelectric point of 10.2).

### Validation of the affinity and specificity of the target DNA sequences

**Figure 5:**
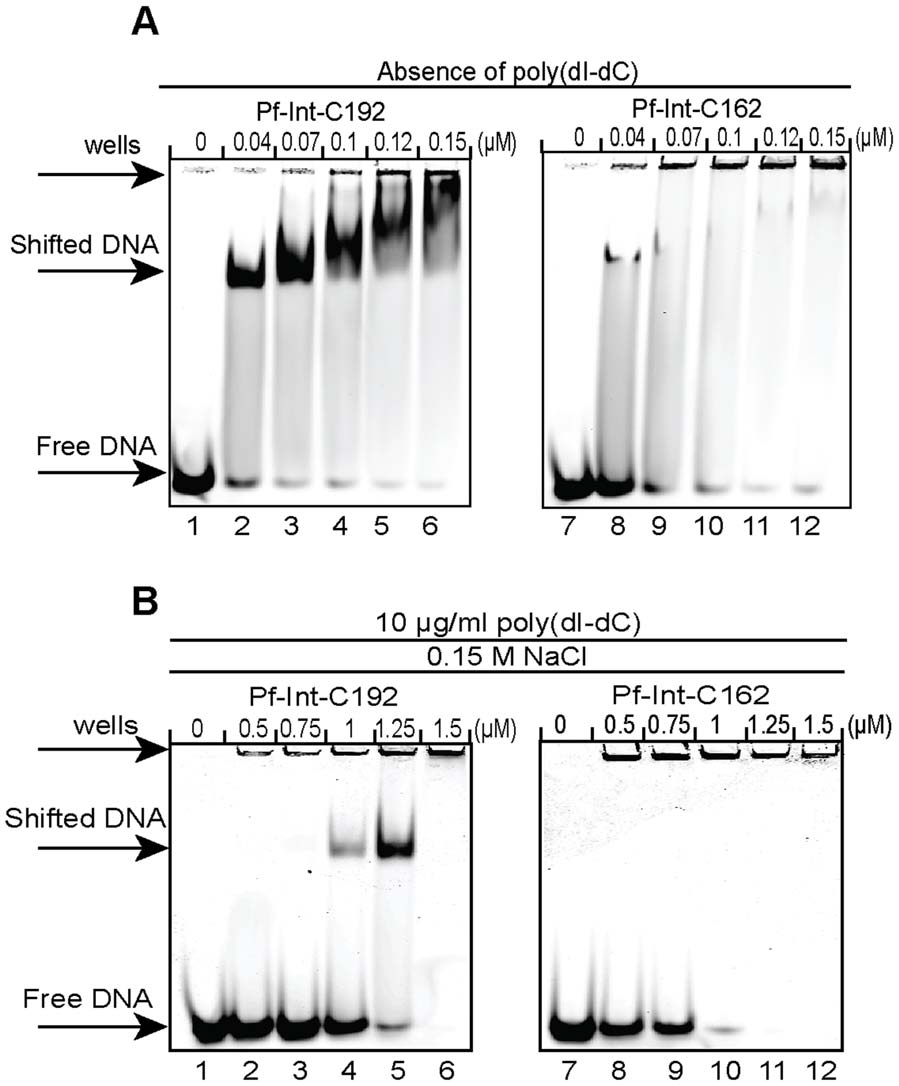
Characterization of Pf-Int interaction with DNA targets. 10 nM of labeled Selex8-DNA were incubated with increasing amounts of Pf-Int-C192 (lanes 1 to 6) and Pf-Int-C162 (lanes 7 to 12) **A**) in absence of poly (dI-dC) and **B**) in the presence of 10 µg/ml of poly (dI-dC).

The interaction of Pf-Int with DNA was characterized by Electrophoretic Mobility Shift Assay (EMSA) using both Pf-Int-C162 and Pf-Int-C192. These experiments showed that Pf-Int is able to form a complex that migrates as a discrete band with the short DNA Selex8 (Figure 5A). EMSAs also demonstrated that the presence of non-specific competitor plays an important role in DNA binding by Pf-Int. Indeed, in the presence of 10 µg/ml poly(dI-dC), the shifted band indicating a protein-DNA complex was only observed with the Pf-Int-C192 protein (Figure 5B, lanes 1 to 6) and not with Pf-Int-C162 (Figure 5B, lanes 7 to 12). The short stretch of amino acids that differs between the two constructs therefore plays an important role in regulating access to the DNA binding site of the recombinase. We noticed that the maximum shift using Pf-Int-C192 (Figure 5B, lane 5) was observed at a ∼15-fold higher molar ratio (protein/DNA) than when the poly(dI-dC) was omitted (Figure 5A, lane 3). The identity of the DNA-binding protein was confirmed by supershift assay using specific antibodies directed either against Pf-Int or a commercial anti-His5 (Figure S4A). Competition mobility shift assays using non-specific DNA competitors confirmed that Pf-Int-C192/Selex 8 interaction is not sequence-specific (Figure S4B and C).

Nonetheless, the Pf-Int/DNA complex can be generated at high ionic concentration, namely 0.9 NaCl (Figure S5A). The binding capacity of Pf-Int-C162 to the identified gDNA targets (fragment 1 shown as a representative) was also tested. As with Selex8, in the presence of poly(dI-dC), only protein/DNA aggregation in the wells was observed (Figure S5B, lanes 1 to 10). Following treatment with proteinase-K, intact DNA was released (Figure S5B, lanes 11 to 13), suggesting that no high molecular DNA species were formed by the action of the recombinase.

## Discussion

Genetic recombination, and in particular site specific recombination, is a basic mechanism for the adaptation of microbial organisms to their hosts or their environment. Interestingly, this process is used as a countermeasure by pathogens to evade the immune system [5]. In the present work we have identified and characterized an ortholog of the tyrosine-recombinase family in the genome of *Plasmodium falciparum.* This gene encodes a 490 aa polypeptide possessing structural features of recombinases.

A comparison of Pf-Int with other Y-SSRs shows a limited degree of conservation at the sequence level, in agreement with previous studies for this family of enzymes. Despite this divergence, these proteins share the same overall structure with two modules corresponding to the N-terminal DNA recognition/specificity and C-terminal catalytic domains, both involved in the binding to the core DNA sequence. For Pf-Int, these two domains include aa ∼190–270, that is attributed to the N-terminal domain of λ-integrase, and aa ∼300–490 that is assigned as a phage integrase-family domain. The latter contains the canonical R-H-R-H/W-Y catalytic residues, with the exception of the first H being replaced by a Y. The same substitution has previously been reported for XisA and XisC from *Anabaena sp.* and SLP1 from *Streptomyces coelicolor* [19]. λ-phage and HP1-phage integrases are considered as bipartite DNA-binding as they interact simultaneously to core DNA and, using an arm-binding domain, to flanking arms DNA sites. Indeed, homology-threaded modeling on the λ-integrase crystal structure predicts that a module homologous to this domain may exist in Pf-Int, spanning from ∼aa 117 to 156. In addition, the segment between the “Arm-binding” module and the N-terminus bears a short stretch that could functionally correspond to the “coupler” for λ-Int (aa 157–191). Sequence alignments of Pf-Int and its Plasmodial homologs (Figure S1A) showed the first 150 aa to be variable. This suggests that this region is less functionally constrained or that it has diverged and adapted to the different species.

Pf-Int mRNA was found to follow a specific expression profile. However, neither our assays nor a global analysis using mass spectrometry [23] detected the Pf-Int protein. This observation is not singular, since 37% of proteins in *P. falciparum* cannot be detected, although their mRNA is produced [23]. Given the fact that Pf-Int RNA levels are not particularly low (Figure S2A), we assume that a post-transcriptional regulation mechanism may act for this gene. It is not surprising as proteins involved in the key DNA transactions like DNA replication, repair, or recombination need to be strongly regulated due to the nature of their activity and their potential impact on the integrity of the genome [24].

Disruption of Pf-Int by a double cross-over showed that the protein is not essential at the intraerythrocytic stage, at least under *in-vitro* culture conditions. We also show that Pf-Int is not indispensable for UV induced DNA-damage repairing. Therefore, Pf-Int could play a role in other life cycle stages within the human host or in the mosquito. The Pf-Int-KO will be of interest for future studies that aim at analyzing the evolution of the subtelomeric variant protein repertoire or other target genes.

Identification of the potential DNA targets of Pf-Int reported in this study using either SELEX or gDNA yielded sequences from a large number of genes. The absence of the potential arm-binding domain in Pf-Int-C162 probably oriented the outcome of the DNA target towards the identification of the core-region sequences only. It is surprising to note that the well represented elements (39% in the case of gDNA) came from the multi-family genes encoding proteins trafficked to the iRBC surface, namely PfEMP1, RIFIN and STEVOR. Moreover, more precise analysis of the gDNA sequences retained showed that they localize partially or totally in exons coding for protein regions that may potentially be under immune pressure. Other multi-copy gene families which are not necessarily surface exposed, and presumably not under immune selection pressure, include PHIST (66 members), and eTRAMP (15 members) [25]. These, among others, are present in the 149 identified sequences obtained by chip hybridization. However, they are far from being present at the same ratio as the variant surface gene members. The traditional core DNA sites are well-defined short sequences with palindromic symmetry. Nevertheless, we were not able to find a conserved motif among the identified DNA targets of Pf-Int. Although it is known that the N-termini of the Y-SSRs (present in Pf-Int-C162) contribute to their specificity [13], more relevant physiological targets might be identified by using the full-length protein in future SELEX experiments. The identified sequences could contain partial sequence match to the ideal target for Pf-Int, therefore pointing to its contribution to a wider impact on plasticity. Alternatively, the repetitive and low complexity sequences captured could lead to a high tendency to form stem loop structures [26]. If the retained gDNA sequences are considered in their single stranded DNA form, they could have the potential to generate stem-loop structures. Such unconventional DNA substrates are not foreign to site-specific recombination reactions, as integron integrases use folded single stranded DNAs from symmetric patches to move cassettes [27].

Our EMSA results showed that Pf-Int has the capacity to bind to Selex8. Based on the susceptibility of the protein/DNA complex to poly(dI-dC) and other non-specific competitors, the interaction seems to be not sequence-specific. Besides the absence of an additional domain in the recombinant proteins, it is also possible that the selectivity could have been altered as a result of the protein expression system used (*E. coli*) that lacks the post-translation modification machinery present in *P. falciparum*. Surprisingly, the complex could be generated at high ionic concentration.

Given that malaria parasite's survival depends on the continual generation of genetic diversity, a mechanism that favors this process has most likely been selected to promote immune evasion. *P. falciparum* could benefit from the presence of a recombinase to promote the generation of variance in the genome. Pf-Int is therefore an appealing candidate that could participate in such an activity. An additional consideration arises if the Pf-Int mechanism resembles that of λ-Int, which is the requirement for the presence of accessory DNA-binding proteins like IHF, Xis and Fis. These accessory proteins can promote the recombination reaction by interacting with different sites on the DNA substrate. The presence of a member of the IHF/HU family has already been reported in *P. falciparum* [28].

In conclusion, our data suggest that Pf-Int is a new member of the tyrosine recombinase family. The existence of a eukaryotic YSSR member in the protozoan parasite *P. falciparum* presents now a testable hypothesis that Pf-Int enhances genetic diversity by catalyzing DNA rearrangements.

## Materials and Methods

### Parasites culture

*P. falciparum* blood stage parasites from the 3D7 strain [29] were cultured using modifications to the method described by Trager and Jensen [30]. Parasites were grown in O+ human erythrocytes in RPMI 1640 medium containing l-glutamine (Invitrogen) supplemented with 5% v/v human serum (PAA Laboratories GmbH) and 5% v/v Albumax II (Invitrogen) in a gas environment of 5% CO2, 5% O2 and 90% N2. Synchronization of cultures consisted of two consecutive 5% sorbitol (Sigma) treatments [31]. We estimated that the parasites were synchronized within a window of approximately 6 h by giemsa staining. Red blood cells were obtained from the Etablissement Français du Sang of Necker hospital, Paris, under agreement with Institut Pasteur, and following guidelines for informed consent of donors for the use of blood or its derivatives for research purposes.

### Life cycle stage mRNA expression monitoring of Pf-Int

RNA preparation and quantitative real-time PCR (qRT-PCR) were performed from synchronized *P. falciparum* blood stage parasite from the 3D7 strain [29]. Total RNA was prepared following the protocol by Kyes et al. [32]. Briefly, infected red blood cells (iRBCs) were washed in PBS, permeabilized with 0.05% Saponin and preserved in Trizol (Invitrogen) at −80°C until use for the isolation of RNA. Genomic DNA contaminants were eliminated using a DNA-free kit (Ambion, Applied Biosystems France). The DNA-free RNA was reverse transcribed with SuperscriptII reverse transcriptase (Invitrogen) and primed with oligo-dT primers (Invitrogen). For each c-DNA synthesis reaction a control was performed without reverse transcriptase.

qRT-PCR was done in a Realplex4 EpgradientS thermal cycler (Eppendorf). Primers (IntegraseF1 and IntegraseB1) were designed such that amplification products would be less than 200 bp in size. The *P. falciparum seryl-tRNA synthetase* gene (*PF07_0073*) was used as internal control. Reactions were performed in 20 µl volumes using SYBR Green PCR master mix (Applied Biosystems) with 200 nM primers, and cycling parameters according to manufacturer's instructions.

Data were acquired at the end of the elongation step of each cycle. Melting-curve analysis was done at the end of each run to validate specificity of the amplification. The specificity was further ascertained by the presence of a single band on an ethidium bromide-stained gel. Additional controls with water only and no reverse transcriptase were included in each run. Each gene was analyzed in duplicate using RNA from two independent RNA preparations.

The transcription level of each gene was assessed as relative copy number to the housekeeping gene *seryl-tRNA synthetase*, and to the expression in ring stage by the ΔΔCt method [33].

### Pf-Int knock-out in *P. falciparum*

Gene knock-out was performed by double cross-over homologous recombination in the *P. falciparum* 3D7 strain. We used the pCC1 plasmid in which two fragments of the coding sequence of Pf-Int gene were inserted on both sides of the human dihydrofolate reductase (hdhfr) resistance cassette. The two Pf-Int gene fragments correspond to 1 kb at the 5′ and 0.8 kb at the 3′ ends of the coding sequence. These were amplified by PCR using the primers IntATG5′/IntATG3′ and IntTAA5′/IntTAA3′ respectively. The pCC1 plasmid also contained the negative selection marker *Saccharomyces cerevisiae* deaminase/uracil phosphoribosyltransferase (ScFCU). Ring-stage parasites were transfected by electroporation with 100 µg of plasmid and selected with 10 nM of WR99210 (Jacobus Pharmaceutical Co. Inc.) as described previously [34]. 4 µM ganciclovir was then used as a negative drug selection to isolate the parasites where double cross-over recombination events had occurred. Resistant parasites were cloned by limiting dilution and analyzed by PCR using primers IntATG5′, IntTAA5′, Chr13.IntF, Chr13.IntR, hDHFR5′, hDHFR3′; pTKATG5′, and pTKATG3′, and Northern blot in order to confirm that the gene had been disrupted.

### Parasite life cycle analysis

Cultures of the 3D7 wild-type and the Pf-Int-KO parasites were synchronized by sorbitol treatment. Cultures at ring stage were dispensed in duplicate into 24-well plates in 0.64 ml aliquots at 5% hematocrit. Every 3 h, 10 µl of mixed culture were removed and the parasites were fixed by addition of 100 µl PBS-0.25% glutaraldehyde and stored at 4°C. The pellet was then washed with 100 µl PBS-50 mM NH_4_Cl and 100 µl of PBS-10× SYBR Green (Invitrogen, S7563) were added. The distribution of stages and parasitemia of each sample were analyzed using BD Biosciences FACSCalibur flow cytometer. 30 000 erythrocytes were counted for each sample. Data were analyzed using FlowJo software (Treestar Inc.) and the points were fitted to an exponential sine wave function using GraphPad Prism (GraphPad Software Inc).

### Response to UV-induced DNA damage

Cultures of the 3D7 wild-type and the Pf-Int-KO parasites were synchronized by sorbitol treatment. In the next cycle, 66 µl of ring-stage infected red blood cells at 2.5% parasitemia were dispensed into 60 cm^2^ petri dishes at 2.2% hematocrit. DNA damage was induced by irradiation with a 254 nm light (Appligene Oncor, UVP England) at 150 and 300×100 µJ/cm^2^. After irradiation the medium was changed and the cultures were diluted to 1.3% parasitemia with fresh red blood cells. Parasitemia was determined daily by flow cytometry as described above.

### Cloning and expression of the *P. falciparum* integrase Pf-Int

The primer pf-int-008-corr was used to obtain c-DNA from a cycle of reverse transcriptase reaction on a wt-3D7 total m-RNA library (Generous gift from Dr Serge Bonnefoy, Institut Pasteur). The resulting c-DNA was then used in a PCR reaction with primers pf-int-010 and pf-int-008-corr in order to amplify the gene segment corresponding to Met162-Ile490, termed as Pf-Int-C162. Restriction sites NcoI and BamHI (primer pfint-top-013, primer pfint-bot-12) were added to the 5′ and 3′ end respectively of the 1,000 bp fragment obtained by PCR, and ligated into pET-43a vector DNA (Novagen, Merck Chemicals France) after restriction digestion with the corresponding enzymes. A sub-fragment containing the aa Leu192 to Ile490 (Pf-Int-C192) was also cloned into pET-43a vector using the primers pfint-c192-top and pfint-490-bottom. The two resulting subsets of Pf-Int include the C-terminal region containing the active site and were used for subsequent assays.

Recombinant proteins were over-expressed in *E. coli* Bl21De3 Codon plus –RIPL cells (Stratagene). The bacterial culture was grown in 800 ml Luria Bertani (LB) media supplemented with 50 µg/ml ampicillin and 25 µg/ml chloramphenicol, in 2 l flasks and shaking at 37°C. Protein expression was triggered with 1 mM Isopropyl-thio-β-D-galactopyranoside (IPTG) after the optical density at 600 nm (OD_600 nm_) reached 0.8, and incubated for 5 hours at 37°C. Harvested cells were frozen at −80°C in buffer A (50 mM Tris-HCl pH 8, 1 M NaCl, 5 mM imidazole, 10% glycerol, 0.1% triton X-100 , and 1 µM Phenyl-methyl-sulfonyl fluoride (PMSF)).

### Recombinant Pf-Int purification

Frozen bacterial cells were thawed to room temperature under cold tap water, and lysed by passage through an Emulsiflex C-5 cell breaker (Avestin Europe GmBH) at 13,000 psi at 4°C. The cell lysate was centrifuged at 14,000 rpm for 40 minutes in an RC-5C using an ss-34 rotor (Sorvall, ThermoScientific). The supernatant was passed through a 0.45 µm filter (Minisart, Sartorius) prior to loading onto a HisTrap HP 5 ml column (Amersham biosciences, GE Lifesciences), and washed with buffer A until baseline (A_280 nm_). The protein was eluted using buffer B (buffer A, with 500 mM NaCl and 250 mM imidazole instead) gradient. Pooled fractions were loaded onto a HiTrap Heparin HP 5 ml column (Amersham biosciences, GE Lifesciences), equilibrated with buffer C (50 mM Tris-HCl, 500 mM NaCl, 10% glycerol, 1 µM PMSF). The protein was eluted using buffer D (buffer C, with 1 M NaCl instead) gradient. When necessary the His-tag was cleaved off by incubating the fractions with TEV-protease (ratio 1∶10, TEV∶protein) and dialyzed over-night at 4°C against 1 l of buffer A. The mixture was then loaded onto a HisTrap HP 5 ml, and fractions containing the cleaved protein were concentrated and dialyzed over-night at 4°C against 1 l of buffer E (50 mM Tris-HCl, 500 mM NaCl, 5% glycerol). The dialyzed protein was loaded on a Superdex G75 (Amersham biosciences, GE Lifesciences) column equilibrated with buffer E. Aliquots of >95% purified protein, as judged by SDS-PAGE and dynamic light scattering, were stored at −80°C in 50 mM Tris-Cl pH 8, 1 M NaCl, 30% glycerol or used immediately as such (stored at 4°C).

### Far-UV circular dichroism and thermal denaturation-CD spectroscopy

CD spectra of Pf-Int variants, at 0.5 mg/ml (13 µM, A_280 nm_ = 0.7), which have been dialyzed over-night at 4°C against repeated changes of buffer (25 mM sodium-phosphate pH 7, 500 mM NaF), were obtained at room temperature on a model 215 AVIV CD spectrophotometer (AVIV biomedical Inc, NJ USA) using a 0.02 cm path length cell. Five successive scans were averaged after subtraction of the background spectra of the sample buffer that has been acquired under identical conditions. The resulting corrected CD intensities were then converted to Δε per residue units. Secondary structure contents were estimated from the far-UV CD spectra using the CDPro package [35], and were averaged from the comparison with three different databases. The stability of Pf-Int was determined by measurements for thermal denaturation at 208 nm in the same instrument. Temperature scans were executed from 10 to 95°C with 1°C/min increments.

### Sedimentation velocity assay for Pf-Int-C162

Pf-Int-C162 samples (24 , 7 et 2 µM) in 20 mM Tris-HCl, pH 8, 200 mM NaCl were centrifuged at a speed of 42,000 rpm in a Beckman Coulter ProteomeLab XL-1 analytical ultracentrifuge at 20°C using a balanced An-60 Ti rotor. Detection of the protein concentration as a function of radial position and time was performed by optical density measurements at a wavelength of 276 nm. Sedimentation velocity data analysis was performed by continuous size distribution analysis c(s) using the program Sedfit 12.1 [36]. The partial specific volume of Pf-Int-C162 (0.7481 ml g^−1^) was estimated from its amino acid sequence using the software Sednterp. The same software was used to estimate buffer viscosity (η = 1.0259 cP) and density (ρ = 1.00704 g ml^−1^).

### SELEX DNA selection by Pf-Int

The search for a target DNA sequence involved an initial library construction using synthetic oligonucleotides followed by affinity selection and amplification of retained sequences (SELEX: Systematic evolution of ligands by exponential enrichment) [37], [38] on Ni-NTA bound His-tagged Pf-Int-C162. The template single stranded DNA sequence library used is a 25 bp random fragment (N)_25_ flanked by two 20 bp amplification primer sites 5′ ATG TCA TAT GGG ATT CGT CG (N)_25_ GCA CCA TAT GCC ATG GAC TC 3′ (Selex4-pfint). The template DNA was synthesized at the 10 OD (A_260 nm_) scale and HPLC purified (Proligo, Sigma Proligo France). The complementary strand for generating the double stranded (ds-DNA) library was synthesized by 1 cycle of PCR using the flanking complementary primer Selex2-pfint and Pfu-turbo DNA polymerase (Stratagene, Agilent Technologies). The double-stranded DNA (dsDNA) library was subsequently amplified by PCR to form the starting stock of dsDNA using primers Selex1-pfint and Selex2-pfint (PCR parameters: 30 cycles, 50.4°C, 72°C, and 94°C annealing, extension and melt temperatures respectively). The DNA was precipitated in 100% ethanol, 0.3 M sodium acetate pH 5.4 at −20°C for 1 hour, centrifuged at 13,200 rpm (Eppendorf 5415 R) for 30 minutes at 4°C, washed with 70% ethanol, and the pellet after speed-vac drying, resuspended in 50 mM Tris-HCl pH 7.8, 0.1 mM EDTA. The efficiency of the detection of SELEX products was tested by amplification of serial dilutions of the library from 1/10, 1/100, 1/1000, 1/10,000, 1/100,000, and 1/1,000,000.

In order to perform the DNA binding, selection, elution and amplification of the SELEX target library, His_6_-Pf-Int-C162 was dialyzed against 50 mM Tris-HCl pH 8.0, 150 mM NaCl, and 10% glycerol overnight to remove excess NaCl. The binding reaction was composed of 7.5 nmoles of DNA and 7.5 nmoles of His_6_-Pf-Int-C162 incubated at 25°C for 30 minutes. A 600 µl protein/DNA mix was loaded on a pre-equilibrated Ni-NTA spin column (Qiagen) (50 mM Tris-HCl pH 8.0, 150 mM NaCl, and 5% glycerol), and spun at 2,000 rpm (Eppendorf 5415 R) for 2 minutes. The flow-through was reloaded to promote complete binding. Elution was carried out in a series of washes containing 250 mM, 450 mM, 550 mM, and 1 M NaCl respectively, in 50 mM Tris-HCl pH 8, 5% glycerol. A final wash using 1 M NaCl, 200 mM imidazole, 50 mM Tris-HCl pH 8, and 5% glycerol was used to remove the stationary phase that included any DNA bound and not released by the recombinase. 5 µl fractions of the eluates were PCR amplified using primers Selex1-pfint and Selex2-pfint with Pfu-turbo polymerase, in 34 cycles at 50.4°C, 72°C, and 94°C annealing, extension and melt temperatures respectively. This procedure was repeated 5 more times, using as starter DNA the highest NaCl eluted fraction as detectable by PCR in the previous cycle; which was at 350 mM, 425 mM, 465 mM, 470 mM and 495 mM NaCl respectively. The final round of selected DNAs (eluted at 750 mM NaCl) was A-tagged using Platinum Taq (Invitrogen) and dATP. The tagged-DNA library was ligated into pCR2.1 vector (Invitrogen) containing a T-overhang, and then transformed into INVFαF' competent cells (Invitrogen) using standard procedures. Blue/white selection was used to identify positive clones for the cells grown on LB plates supplemented with X-Gal 80 µg/ml and ampicillin 100 µg/ml. 60 positive colonies were picked, midi-prepped (Qiagen), and the plasmid DNA sent for sequencing using the M13 reverse and M13 forward sites (Genome-express, Cogenics, Beckman-Coulter Genomics France). *Mycobacterium tuberculosis* fumarate hydratase, a non-DNA binding protein, (Generous gift, Dr Ahmed Haouz, Institut Pasteur) was used to monitor non-specific binding.

### Genomic DNA target selection by Pf-Int

Genomic DNA (g-DNA) from *P. falciparum* 3D7 was sonicated (Bioruptor, Diagenode, Belgium) in order to yield ∼200–300 bp fragments at 110 µg/ml. The sonicated DNA was run on an agarose gel and the band corresponding to a 200–300 bp fragment extracted and purified using a gel extraction kit (Qiagen). The ends of the DNA fragments were tagged with adaptors from the whole genome amplification Genoplex kit (WGA2, Sigma-Aldrich) following the manufacturer's instructions. Briefly, a supplied oligonucleotide library composed of a proprietary known and partly randomized ends is used to match and ligate with the variable sonicated DNA ends. Supplied primers complementary to the known regions of the adaptor are then used to PCR amplify and standardize the library.

DNA selection was carried out using the affinity-fixed His_6_-Pf-Int-C162 on Ni-NTA beads (Qiagen). The beads were equilibrated with two 500 µl volumes of buffer composed of 50 mM Tris-HCl pH 8, 50 mM NaCl. They were loaded with 1.1 µg/ml sonicated gDNA and 1.3 µg/ml of His_6_-Pf-Int-C162 in equilibration buffer. The beads were washed 5 times with the same buffer until no DNA could be detected (A_260 nm_) in the eluate (Nanodrop, Thermo Fisher). The DNA was eluted in successive steps using 250 mM to 750 mM NaCl in equilibration buffer in increments of 50 mM. A final elution with 250 mM imidazole was used to remove any His_6_-Pf-Int-C162 to which tightly bound DNA might have been attached. Controls using DNA alone (no Pf-Int) or *Mycobacterium tuberculosis* fumarate hydratase, a non-DNA binding protein, were used to monitor non-specific binding.

After elution, the highest NaCl fraction (750 mM) and imidazole eluted DNA were PCR amplified using the primers provided in the WGA2 kit. The amplified DNAs were ligated into pCR-BluntII-topo vector (Invitrogen), and transformed into Top10 competent cells (Invitrogen) grown on LB supplemented with kanamycin at 50 µg/ml. Midi-prepped DNAs (Qiagen) were sequenced (Genome-express, Cogenics, Beckman-Coulter Genomics France) to determine the composition of the retained DNAs. The DNAs were identified by BLAST [39] against a 3D7 *P. falciparum* genomic database.

### Microarray analysis of potential DNA targets of Pf-Int

As an alternative to sequencing, which could only cover the number of colonies picked for sequencing and therefore not provide a more complete repertoire of bound DNA, we used a microarray hybridization approach in order to identify the DNA fragments bound by Pf-Int. After elution, the highest NaCl fraction (750 mM) and imidazole eluted Pf-Int bound DNA were PCR amplified through 3 rounds of 30 cycles to about 4 µg of material. The DNA was submitted for hybridization using a custom designed array composed of *P. falciparum* 200-bp-spaced genomic library (Roche Nimblegen Inc, Europe) [22]. DNA was labeled using random primers coupled to a fluorochrome and hybridized according to NimbleGen Systems procedures. Each feature on the array has a corresponding scaled log2-ratio. This is the ratio of the input signals for the experimental and test samples that were co-hybridized to the array. The log2-ratio is computed and scaled to center the ratio data around zero. Scaling is performed by subtracting the bi-weight mean for the log2-ratio values for all features on the array from each log2-ratio value. Peak data are generated from the scaled log2-ratio data. NimbleScan detects peaks by searching for 4 or more probes whose signals are above the specified cutoff values, ranging from 90% to 15%, using a 500 bp sliding window. The cutoff values are a percentage of a hypothetical maximum, which is the mean + 6 (standard deviation). The ratio data is randomized 20 times to evaluate the probability of “false positives.” Each peak is then assigned a false discovery rate (FDR) score based on the randomization. Peaks with FDR score between 0 and 0.1 and present in the four replicates were used for the analysis.

### Electrophoretic mobility shift assay using Pf-Int and DNA oligonucleotides

Electrophoretic mobility shift assay (EMSA) experiments were carried out using DNAs that were designed based on the sequences obtained from the SELEX experiment or from the g-DNA selection experiment. Thus, for the SELEX DNAs, 5′-DY682-labeled (Eurofins MWG Operon) and non-labeled (Sigma-Aldrich) DNAs were chemically synthesized as single stranded DNA, HPLC purified and annealed. Selected g-DNA (PlasmoDB formatted Pf3D7_chromosome N°:start bp..end bp, fragment 1 Pf3D7_07:639471..639635, fragment 2 Pf3D7_04:907385..907475, fragment 3 Pf3D7_07:1079071..1079205 and fragment 4 Pf3D7_04:17317..17442) were amplified by PCR from the midi-preps using 5′-IRD700-labeled T7 primers.

EMSA standard reactions contained 10 nM DNA mixed with varying amounts of Pf-Int protein (either Pf-Int-C162 or Pf-Int-C192) from 5 nM to 1.5 µM, in a final volume of 20 µl with binding buffer F (50 mM Tris pH 8, 0.1 mM EDTA, 5 mM DTT, 50 µg/ml BSA, 2% glycerol, 0.5% Tween 20, 150 mM NaCl and 10 µg/ml poly (dI-dC) (Sigma-Aldrich, cat. No. P4929)). When specified some experiments were carried out without poly (dI-dC). After incubation at 20°C for 20 minutes, reactions were mixed with 1 µl of 60% glycerol and analyzed on native polyacrylamide gels (nPAGE). Pre-equilibrated 20-cm×20-cm 5% gels were ran at 10 V/cm for 90 min at 18°C using TBE 0.5× (Biosolve, Netherlands) as running buffer. After electrophoresis, the gel was read on an Odyssey scanner (Li-COR, NE USA) according to the manufacturer's instructions and the resulting image analyzed by the provided software (v. 3.0).

Supershift experiments were carried out using either an antibody raised against the purified recombinant Pf-Int or a commercial anti-His_5_ (Qiagen, 1014992). 10 nM of Selex8 were mixed with the corresponding amounts of purified His_6_-Pf-Int-C192 for 20 minutes at room temperature before adding the antibodies anti-Pf-Int and anti-His_5_ for an additional 20 minutes. In specific cases, the purified Pf-Int was pre-incubated with the anti-Pf-Int for 20 minutes at room temperature before addition of the DNA for an additional 20 minutes.

Evaluation of the well-aggregated complex was performed using proteinase K digestion. Pf-Int-C162:g-DNA (fragment 1 to 4) treatment with proteinase K (Eurobio, France) was carried out after incubating Pf-Int-C162 with 10 nM DNA in the ratio 300∶1 for 20 minutes at 20°C in buffer F, followed by the addition of 5 µg of protease, incubated at 37°C for 30 minutes, and analyzed by nPAGE.

### DNA specificity determination for Pf-Int-C192

Competition binding assays were done using DNA from the SELEX experiment and as competitors either a modified attC integron integrase binding site, fragments of two different *Plasmodium* genes (PfHSP70 (PlasmoDB id. PF08_0054), PfActin-II (PlasmoDB id. PF14_0124)), or a random DNA probe (Table S1). Purified protein (Pf-Int-C192, 0.3 µM) was allowed to bind to 1,500 nM unlabeled competitor or unlabeled Selex8-22 (Table S1) for 20 minutes at 20°C in 10 µl of binding buffer F. 10 nM of labelled Selex8-22 was then added for an additional 20 min for the chase. The residual DNA/protein complex was then separated on nPAGE as described above.

Selectivity experiments were performed using the *P. falciparum* putative transcriptional factor AP2 domain produced as a GST-AP2 domain fusion (PlasmoDB id. PFF0200C protein) (generous gift from Dr Rafael Martins-Miyazawa, Institut Pasteur) and its corresponding DNA target AP2-probe (Table S1) from the 5′UTR of the MAL7P1.119 gene [40]. 10 nM of Selex8 DNA or AP2-probe were mixed with either 1.25 µM Pf-Int-C192 or 20 µM GST-AP2-domain in a final volume of 20 µl of buffer F and incubated for 20 minutes at 20°C. The DNA/protein complex was then separated on nPAGE as described above.

## Supporting Information

Figure S1 **Amino acid sequence alignment of Pf-Int with its homologous.** Clustal alignment of Pf-Int with its homologues present in **A**) *P. vivax*, *P. chabaudii*, *P. bergheii*, *P. knowlesi and P. yoelii*, and **B**) *Toxoplasma gondii* and *Neospora caninum*.
(TIF)

Figure S2 **Expression profile for Pf-Int during life stages of** ***P. falciparum*** **.** **A**) mRNA expression of Pf-Int was examined by real-time PCR at the three parasite living stages: ring, trophozoite and schizont. Values are the mean of two independent experiments whose standard deviation is shown by the bars. Values are expressed as relative to the m-RNA level at ring stage (left panel) and relative to the m-RNA level of the PfSeryl-tRNA synthetase (right panel) and **B**) Protein expression was characterized by Western blot analysis of parasites extracts from different parasite stages. *P. falciparum* gametocytes (lanes 1, 2 and 5), *P. falciparum* rings (lane 3), trophozoites/schizonts (lane 4), *P. berghei* sporozoites (lane 6) and *P. berghei* asexual stages (lane 7). The extracts were run in a 4–20% NuPage gel/MOPS. The anti-Pf-Int antibody raised against the recombinant purified protein was used as a primary antibody at 1/500 dilution.
(TIF)

Figure S3 **Pf-Int knock-out strategy and its validation.** The Pf-Int gene was knocked out by a double cross over reaction in the 3D7 line. **A**) The Pf-Int-KO parasite was created using the plasmid pCC1-Pf-Int and WR99210 and ganciclovir selection. In pCC1-Pf-Int plasmid, the hDHFR resistance cassette (cyan) was inserted between two regions of the Pf-Int gene: Pf-Int box 1 (red) and Pf-Int box 2 (yellow). The pCC1-Pf-Int plasmid also contained the ScFCU selection cassette. **B**) The disruption of the gene was verified by PCR reactions using different combinations of primers pTKATG5′/pTKATG3′(5/6), Chr13IntF/Chr13IntR(1/4), Chr13IntF/hDHFR3′(1/2), hDHFR5′/Chr13IntR(3/4) and IntATG5′/IntTAA3′(7/8). DNAs used as templates are from WT parasite (lanes 1, 5, 7, 9, 11 and 13), Pf-Int-KO parasite (lanes 2, 6, 8, 10 and 12), pCC1 plasmid (lane 4) and pCC1-Pf-Int (lane 3). **C**) The absence of Pf-Int-transcript in the knocked-out parasites was verified by Northern blot using probes against box1, box2 and the replaced fragment (RF). Lane 1: mRNA from WT parasite. Lane 2: mRNA from Pf-Int-KO parasites. A probe against the actin transcript was used as a loading control.
(TIF)

Figure S4 **Analysis of DNA binding by Pf-Int.** **A**) Supershift assay confirms the presence of Pf-Int bound to the DNA. The binding of His_6_-Pf-Int-C192 to Selex8 DNA target was examined by supershift using either an antibody raised against the purified recombinant Pf-Int or a commercial anti-His_5_ in combination with a His-tagged version of Pf-Int-C192 protein. 10 nM of Selex8 were mixed with 0.04 µM or 0.2 µM purified His_6_-Pf-Int-C192 (lanes 3, 5, 7 and 10) and (lanes 4, 6, 8 and 11) respectively. The DNA was pre-incubated with the corresponding amount of Pf-Int for 20 minutes at room temperature before adding the antibodies for an additional 20 minutes (lanes 5, 6, 10 and 11). For lanes 7 and 8, the purified Pf-Int was pre-incubated with the anti-Pf-Int for 20 minutes before addition of the DNA target. Addition of the Pf-Int antibody disrupts the formation of the DNA-Pf-Int complex (see lanes 5–8 in comparison with lanes 3–4: disappearance of shifted band and increase of free DNA). Addition of a commercial anti-His_5_ antibody resulted in the disappearance of the protein-complex band without release of the bound DNA and a tendency to form a higher molecular weight band (see lanes 10–11 compared with lanes 3–4). **B**) Purified GST-AP2 domain (lanes 1 to 4) and Pf-Int-C192 (lanes 5 to 8) were allowed to bind to 10 nM labeled Selex8 probe or AP2 probe in appropriate binding buffer. Lanes 1, 2, 5 and 6: Selex8 probe. Lanes 3, 4, 7 and 8: AP2 probe. Lanes 2 and 4: presence of 20 µM AP2 domain. Lanes 6 and 8: in presence of 1.25 µM Pf-Int-C192. **C**) 0.3 µM of Pf-Int-C192 were incubated with labeled Selex8-22 bp probe (10 nM) in the absence (lane 2) or presence of 150-fold excess of the unlabeled dsDNA competitor. Lane 3: Unlabeled Selex8-22 bp probe. Lane 4: modified (attC) integron integrase binding site. Lane 5: HSP70 DNA. Lane 6: ActinII DNA. Lane 7: random DNA sequence. SC: specific competitor, NSC: non-specific competitor.
(TIF)

Figure S5 **Analysis of DNA binding by Pf-Int.** **A**) The presence of 900 mM NaCl in the binding buffer of Pf-Int-C192/Selex8 was also tested. **B**) 10 nM of labeled fragment 1 gDNA target were allowed to bind with increasing amounts of Pf-Int-C162 (lanes 1 to 10). 10 nM of labeled fragment 1 gDNA target were incubated with 3 µM Pf-Int-C162 for 20 minutes (Lane 12) and then digested with the proteinase-K (Lane 13).
(TIF)

Table S1 **Oligonucleotides used for qRT-PCR, cloning and EMSA assays.** The DNA primers used for cloning and qRT-PCR were single stranded, whereas the ones destined for EMSAs were annealed to their reverse complement to form the corresponding double stranded DNA. The EMSA DNAs, when specified, were uniquely 5′ labeled with DY682 on the strands specified in this table.
(DOCX)

Table S2 **Potential DNA targets identified for Pf-Int.** **A**) Targets identified by sequencing of eluted fragments retained by Pf-Int. **B**) Targets identified by chip hybridization of eluted fragments.
(DOCX)
