# Supplementary figures and images for: Initial Characterization of the Pf-Int Recombinase from the Malaria Parasite Plasmodium falciparum
Source: PLoS One. 2012 Oct 8;7(10):e46507. doi: 10.1371/journal.pone.0046507 (PMC3466309; doi:10.1371/journal.pone.0046507)

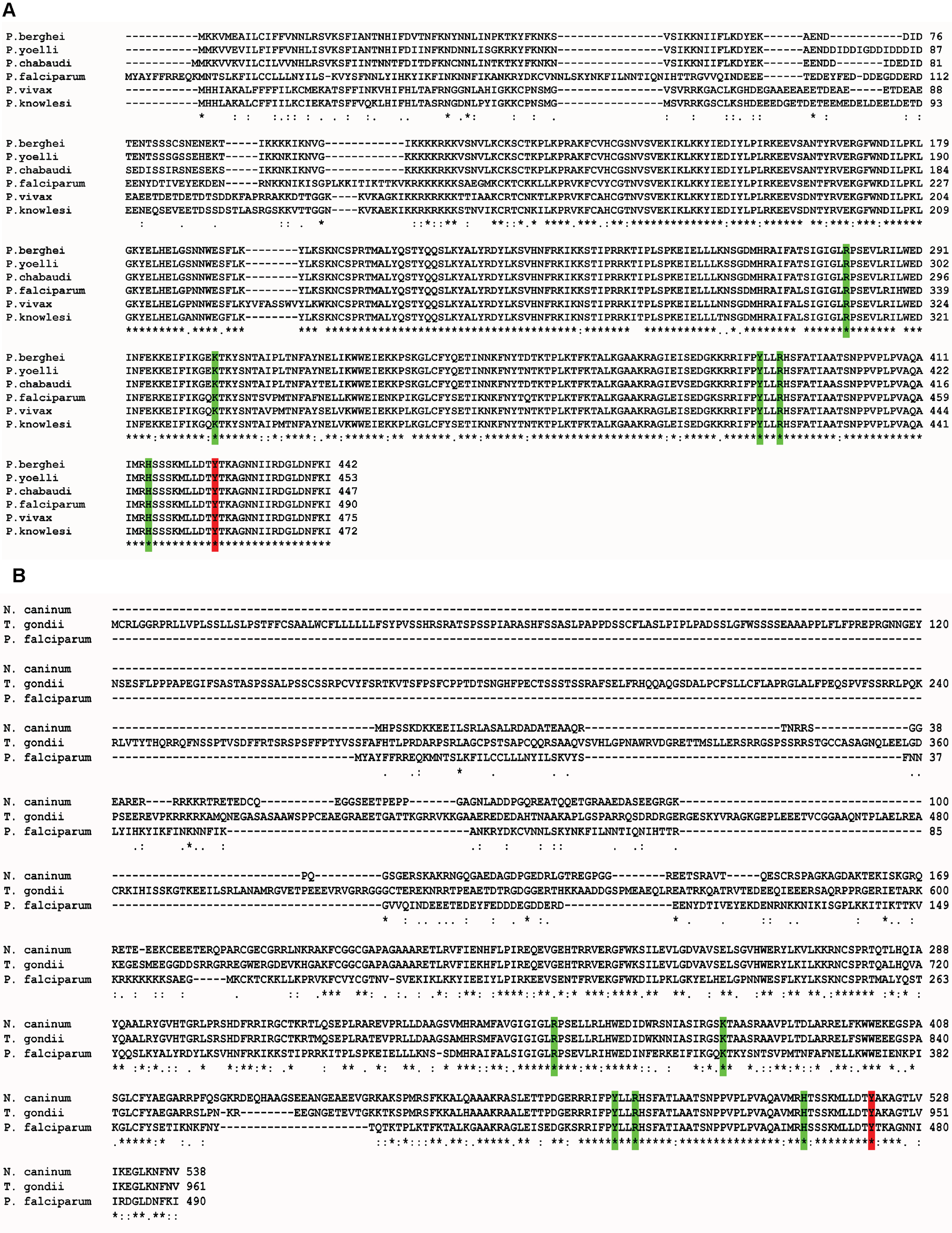

Supplement: Figure S1 — Amino acid sequence alignment of Pf-Int with its homologous. Clustal alignment of Pf-Int with its homologues present in A) P. vivax, P. chabaudii, P. bergheii, P. knowlesi and P. yoelii, and B) Toxoplasma gondii and Neospora caninum. (TIF) [file pone.0046507.s001.tif]

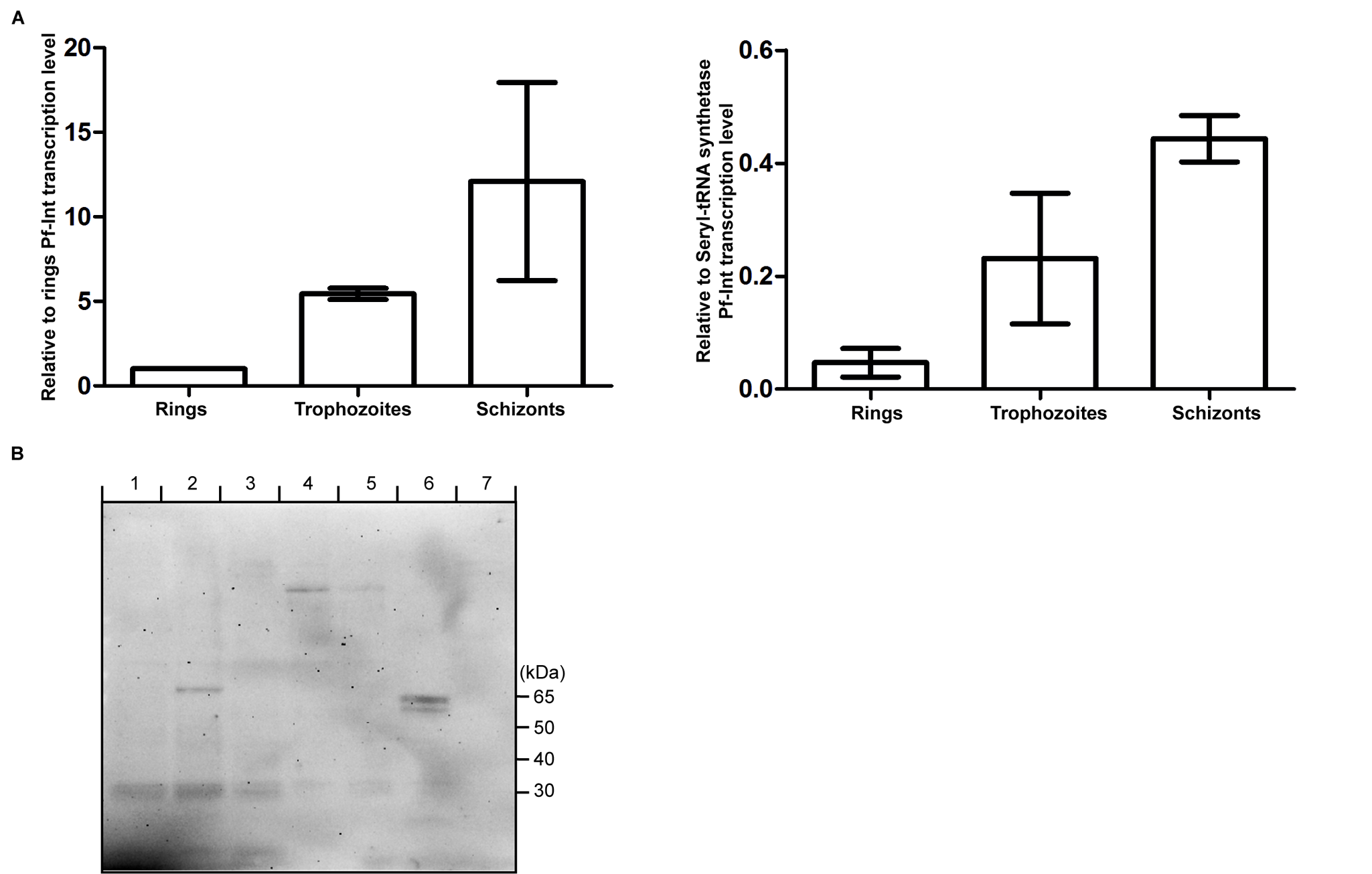

Supplement: Figure S2 — Expression profile for Pf-Int during life stages of P. falciparum . A) mRNA expression of Pf-Int was examined by real-time PCR at the three parasite living stages: ring, trophozoite and schizont. Values are the mean of two independent experiments whose standard deviation is shown by the bars. Values are expressed as relative to the m-RNA level at ring stage (left panel) and relative to the m-RNA level of the PfSeryl-tRNA synthetase (right panel) and B) Protein expression was characterized by Western blot analysis of parasites extracts from different parasite stages. P. falciparum gametocytes (lanes 1, 2 and 5), P. falciparum rings (lane 3), trophozoites/schizonts (lane 4), P. berghei sporozoites (lane 6) and P. berghei asexual stages (lane 7). The extracts were run in a 4–20% NuPage gel/MOPS. The anti-Pf-Int antibody raised against the recombinant purified protein was used as a primary antibody at 1/500 dilution. (TIF) [file pone.0046507.s002.tif]

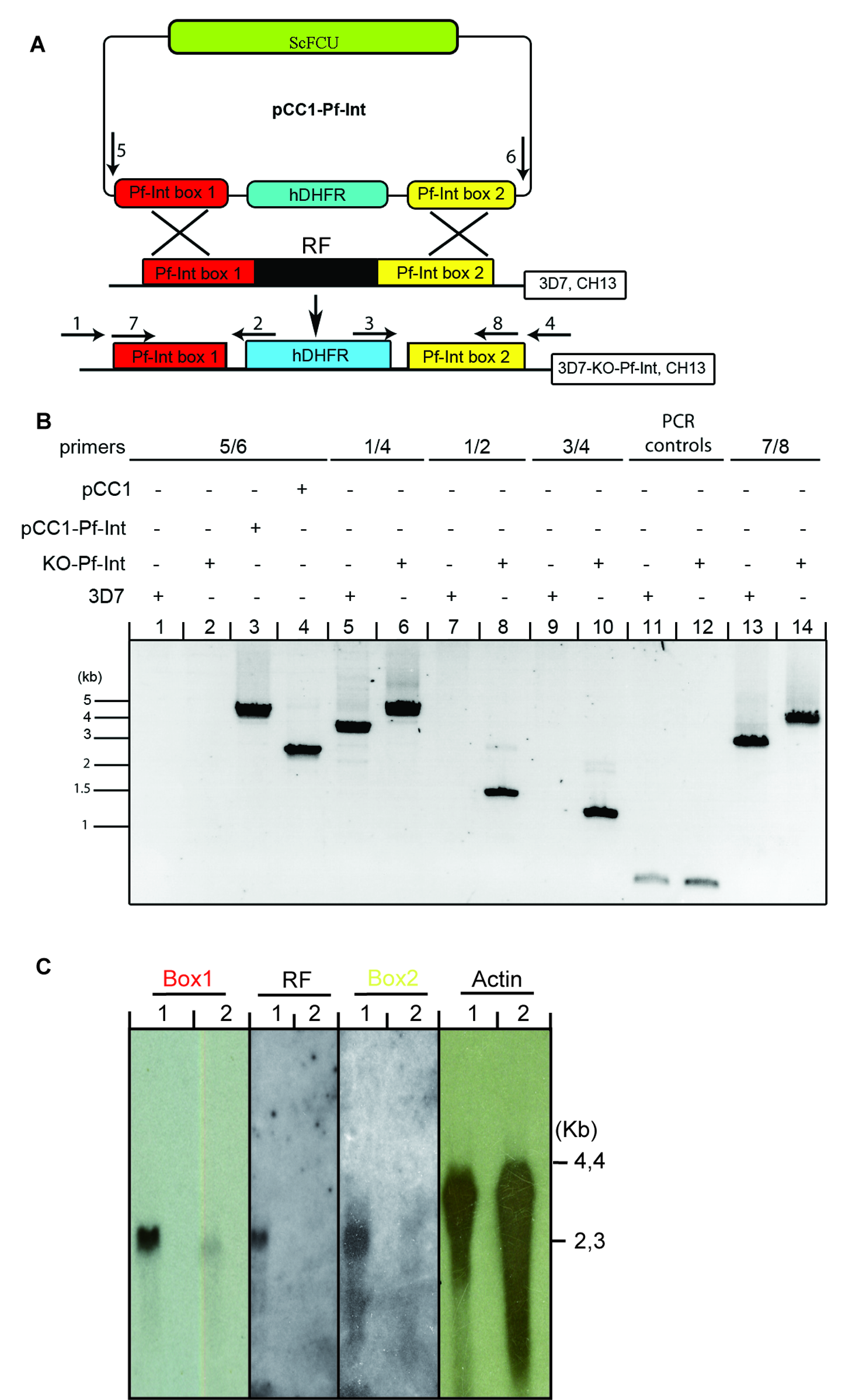

Supplement: Figure S3 — Pf-Int knock-out strategy and its validation. The Pf-Int gene was knocked out by a double cross over reaction in the 3D7 line. A) The Pf-Int-KO parasite was created using the plasmid pCC1-Pf-Int and WR99210 and ganciclovir selection. In pCC1-Pf-Int plasmid, the hDHFR resistance cassette (cyan) was inserted between two regions of the Pf-Int gene: Pf-Int box 1 (red) and Pf-Int box 2 (yellow). The pCC1-Pf-Int plasmid also contained the ScFCU selection cassette. B) The disruption of the gene was verified by PCR reactions using different combinations of primers pTKATG5′/pTKATG3′(5/6), Chr13IntF/Chr13IntR(1/4), Chr13IntF/hDHFR3′(1/2), hDHFR5′/Chr13IntR(3/4) and IntATG5′/IntTAA3′(7/8). DNAs used as templates are from WT parasite (lanes 1, 5, 7, 9, 11 and 13), Pf-Int-KO parasite (lanes 2, 6, 8, 10 and 12), pCC1 plasmid (lane 4) and pCC1-Pf-Int (lane 3). C) The absence of Pf-Int-transcript in the knocked-out parasites was verified by Northern blot using probes against box1, box2 and the replaced fragment (RF). Lane 1: mRNA from WT parasite. Lane 2: mRNA from Pf-Int-KO parasites. A probe against the actin transcript was used as a loading control. (TIF) [file pone.0046507.s003.tif]

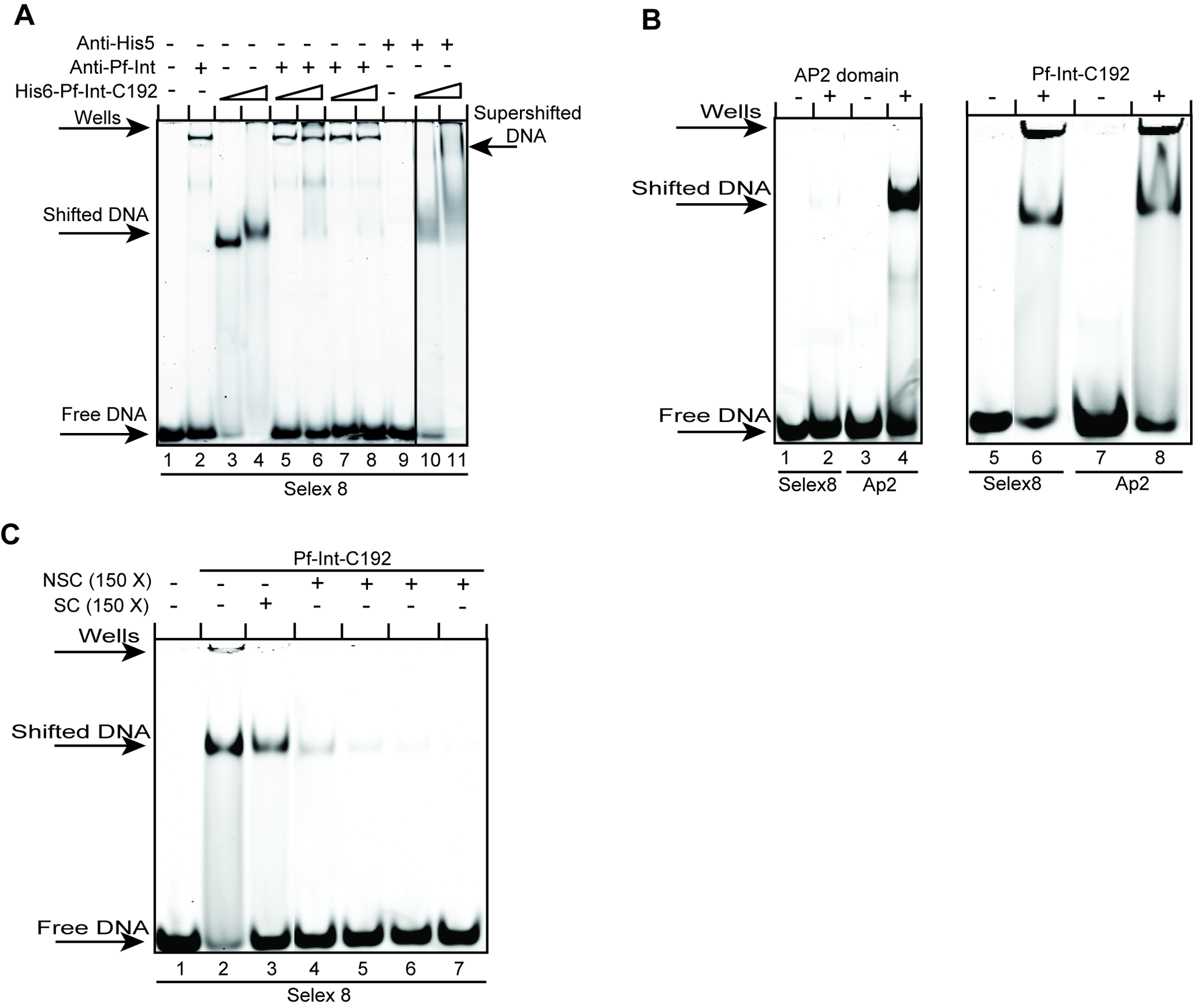

Supplement: Figure S4 — Analysis of DNA binding by Pf-Int. A) Supershift assay confirms the presence of Pf-Int bound to the DNA. The binding of His6-Pf-Int-C192 to Selex8 DNA target was examined by supershift using either an antibody raised against the purified recombinant Pf-Int or a commercial anti-His5 in combination with a His-tagged version of Pf-Int-C192 protein. 10 nM of Selex8 were mixed with 0.04 µM or 0.2 µM purified His6-Pf-Int-C192 (lanes 3, 5, 7 and 10) and (lanes 4, 6, 8 and 11) respectively. The DNA was pre-incubated with the corresponding amount of Pf-Int for 20 minutes at room temperature before adding the antibodies for an additional 20 minutes (lanes 5, 6, 10 and 11). For lanes 7 and 8, the purified Pf-Int was pre-incubated with the anti-Pf-Int for 20 minutes before addition of the DNA target. Addition of the Pf-Int antibody disrupts the formation of the DNA-Pf-Int complex (see lanes 5–8 in comparison with lanes 3–4: disappearance of shifted band and increase of free DNA). Addition of a commercial anti-His5 antibody resulted in the disappearance of the protein-complex band without release of the bound DNA and a tendency to form a higher molecular weight band (see lanes 10–11 compared with lanes 3–4). B) Purified GST-AP2 domain (lanes 1 to 4) and Pf-Int-C192 (lanes 5 to 8) were allowed to bind to 10 nM labeled Selex8 probe or AP2 probe in appropriate binding buffer. Lanes 1, 2, 5 and 6: Selex8 probe. Lanes 3, 4, 7 and 8: AP2 probe. Lanes 2 and 4: presence of 20 µM AP2 domain. Lanes 6 and 8: in presence of 1.25 µM Pf-Int-C192. C) 0.3 µM of Pf-Int-C192 were incubated with labeled Selex8-22 bp probe (10 nM) in the absence (lane 2) or presence of 150-fold excess of the unlabeled dsDNA competitor. Lane 3: Unlabeled Selex8-22 bp probe. Lane 4: modified (attC) integron integrase binding site. Lane 5: HSP70 DNA. Lane 6: ActinII DNA. Lane 7: random DNA sequence. SC: specific competitor, NSC: non-specific competitor. (TIF) [file pone.0046507.s004.tif]

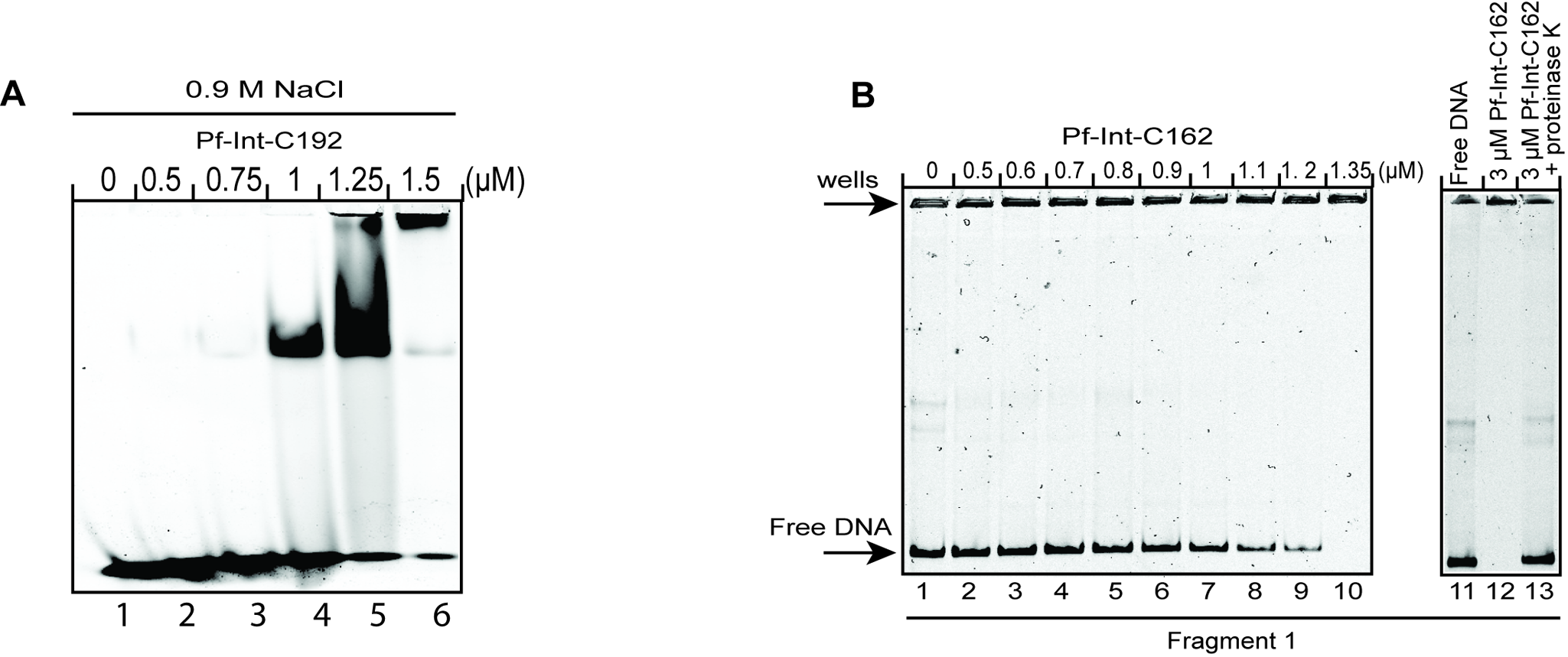

Supplement: Figure S5 — Analysis of DNA binding by Pf-Int. A) The presence of 900 mM NaCl in the binding buffer of Pf-Int-C192/Selex8 was also tested. B) 10 nM of labeled fragment 1 gDNA target were allowed to bind with increasing amounts of Pf-Int-C162 (lanes 1 to 10). 10 nM of labeled fragment 1 gDNA target were incubated with 3 µM Pf-Int-C162 for 20 minutes (Lane 12) and then digested with the proteinase-K (Lane 13). (TIF) [file pone.0046507.s005.tif]
