# Supplementary material for: Initial Characterization of the Pf-Int Recombinase from the Malaria Parasite Plasmodium falciparum
Source: PLoS One. 2012 Oct 8;7(10):e46507. doi: 10.1371/journal.pone.0046507 (PMC3466309; doi:10.1371/journal.pone.0046507)
Supplement: Table S1 — Oligonucleotides used for qRT-PCR, cloning and EMSA assays. The DNA primers used for cloning and qRT-PCR were single stranded, whereas the ones destined for EMSAs were annealed to their reverse complement to form the corresponding double stranded DNA. The EMSA DNAs, when specified, were uniquely 5′ labeled with DY682 on the strands specified in this table. (DOCX) [file pone.0046507.s006.docx]

**Table S1:** Oligonucleotides used for qRT-PCR, cloning and EMSA assays

| **Oligo name** | **DNA sequence** |
| --- | --- |
| pf-int-008-corr | 5’-GAAGTTATCAAGACCATCACGAATAATGTTATTTCC-3’ |
| pf-int-010 | 5’-CTGCTGAAGGGATGAAATGTAAAACATGC-3’ |
| pf-top-013 | 5’ GATACACCATGGCGAAATGTAAAACATGCAAGAAATTATTG 3’ |
| pf-bot-12 | 5’ GCATATGGGATCCTTATATTTTGAAGTTATCAAGACC 3’ |
| pfint-c192-top | 5’-GGTCCATGGCGTTGAAAAAATATATAGAAG-3’ |
| pfint-490-bottom | 5’-GAATTCGGATCCTTATATTTTGAAGTTATCAAGACCATC-3’ |
| Selex2-pfint | 5’-GAGTCCATGGCATATGGTGC-3’ |
| Selex1-pfint | 5’-ATGTCATATGGGATTCGTCT-3’ |
| IntegraseF1 | 5’-ATTGGACTCAGACCATCG-3’ |
| IntegraseB1 | 5’-AGTTTGTCATAGGAACGG-3’ |
| ActinII | 5’-TCTGAAGAAGCTGTTGCTTTAGTAGTTGATAATGGAAGTGGT-3’ |
| Hsp70 | 5’-GCTAGTGCAAAAGGTTCAAAACCAAATTTACCAGAATCCAAT-3’ |
| Selex8 | 5’-CAAACACACTGTGCGCGCGTCG-3’ |
| Selex8-22 | 5’-ACCCAAACACACTGTGCGCGCGTCG-3’ |
| Modified attC site | 5’-GGATCCGGTTATAACGAACGCCTAAGGGGCTGTCAACGCA-3’ |
| Random probe | 5’-GTACTTACTCTAATCTAGTCGTAGTATGTATGCCTCAATGAA-3’ |
| AP2probe | 5’-AAATAAGTGTGTGCACATGTAACCA-3’ |
| IntATG5’ | 5‘-CCCCGCGGATGTATGTATATATATATATGTATGC-3’ |
| IntATG3’ | 5’-GGACTAGTGGACCCAATTCGTGCAGTTC-3’ |
| IntTAA5’ | 5’-CGGAATTCTGGGAAATAGAAAATAAGCCCATT-3’ |
| IntTAA3’ | 5’-CGCCCTAGGTTATATTTTGAAGTTATCAAGACCA-3’ |
| Chr13.IntF | 5’-GAGAAGTTATTAATTTGGTGAAAG-3’ |
| Chr13.IntR | 5’-CATATTAGAAACGTCCAAATAAAT-3’ |
| pTK ATG 5’ | 5’-CTTTAAATTCATGCAAAAATTTAC-3’ |
| pTK ATG 3’ | 5’- CCAATAGATAAAATTTGTAGAG-3’ |
| hDHFR 5’ | 5’-GCATGGTTCGCTAAACTGCATC-3’ |
| hDHFR 3’ | 5’-TCATTCTTCTCATATACTTC-3’ |
