# Supplementary material for: Initial Characterization of the Pf-Int Recombinase from the Malaria Parasite Plasmodium falciparum
Source: PLoS One. 2012 Oct 8;7(10):e46507. doi: 10.1371/journal.pone.0046507 (PMC3466309; doi:10.1371/journal.pone.0046507)
Supplement: Table S2 — Potential DNA targets identified for Pf-Int. A) Targets identified by sequencing of eluted fragments retained by Pf-Int. B) Targets identified by chip hybridization of eluted fragments. (DOCX) [file pone.0046507.s007.docx]

**Table S2A:** Genomic DNA targets identified by sequencing

| **Chromosome** | **Position** | **Function** |
| --- | --- | --- |
| 3 | 3320 to 3149 | Intergenic region |
| 4 | 62160 to 62238 | Pfemp1 |
| 4 | 907385 to 907475 | zinc finger protein, putative |
| 4 | 17317 to 17442 | Close to pfemp1 |
| 4 | 821245 to 821093 | conserved Plasmodium protein, unknown function |
| 4 | 749329 to 749184 | conserved Plasmodium protein, unknown function |
| 5 | 454262 to 454163 | Intergenic region |
| 5 | 410817 to 410669 | phosphatidylinositol 4-kinase, putative |
| 5 | 655247 to 654983 | metabolite/drug transporter, putative |
| 6 | 1131148 to 1131322 | HECT-domain (ubiquitin-transferase), putative |
| 6 | 183039 to 182898 | conserved Plasmodium protein, unknown function |
| 7 | 639471 to 639635 | PFEMP1 |
| 7 | 1079071 to 1079205 | mitochondrial import inner membrane translocase subunit tim14 |
| 8 | 286575 to 286466 | Intergenic region |
| 9 | 19719 to 19994 | Close to pfemp1 |
| 9 | 289122 to 288998 | conserved Plasmodium protein, unknown function |
| 10 | 1482876 to 1482970 | conserved Plasmodium protein |
| 13 | 1810792 to 1810678 | Intergenic region |
| 14 | 2783657 to 2783724 | conserved Plasmodium protein, unknown function |
| 14 | 1991086 to 1990956 | chloroquine resistance marker protein |
| 14 | 2353468 to 2353589 | conserved Plasmodium protein, unknown function |
| 14 | 3074965 to 3074715 | Intergenic region |

**Tables S2B**: Genomic DNA targets identified by Chip analysis

| **Chromosome** | **Position** | **Function** |
| --- | --- | --- |
| chr$1 | 51450 to 51690 | Rifin |
| chr$1 | 60690 to 61040 | Rifin |
| chr$1 | 350430 to 350730 | Conserved protein unknown function |
| chr$1 | 478050 to 478290 | rrna encoding |
| chr$1 | 556710 to 557130 | Close to pfemP1 |
| chr$1 | 599620 to 599720 | Close to Stevor |
| chr$2 | 33560 to 33860 | Rifin |
| chr$2 | 54400 to 54740 | Stevor |
| chr$2 | 787000 to 787500 | PHIST domain protein |
| chr$2 | 889860 to 890280 | Rifin |
| chr$2 | 896800 to 897050 | Rifin |
| chr$2 | 902500 to 902560 | Conserved protein |
| chr$2 | 913650 to 914200 | Rifin |
| chr$3 | 58800 to 59200 | Rifin |
| chr$3 | 122460 to 122730 | pfemP1 pseudo gene |
| chr$3 | 191090 to 191170 | IBR domain protein |
| chr$3 | 232920 to 233070 | Conserved protein unknown function |
| chr$3 | 233670 to 233730 | Conserved protein unknown function |
| chr$3 | 233970 to 234030 | Conserved protein unknown function |
| chr$3 | 234270 to 234330 | Conserved protein unknown function |
| chr$3 | 1012980 to 1013580 | Rifin |
| chr$3 | 1015920 to 1016340 | Rifin |
| chr$3 | 1024920 to 1025160 | Rifin |
| chr$4 | 38100 to 38700 | pfemP1 |
| chr$4 | 39300 to 39900 | pfemP1 |
| chr$4 | 40200 to 40800 | pfemP1 |
| chr$4 | 56700 to 57000 | pfemP1 |
| chr$4 | 58200 to 58800 | pfemP1 |
| chr$4 | 60600 to 60900 | pfemP1 |
| chr$4 | 86300 to 86800 | Rifin |
| chr$4 | 88900 to 89500 | Rifin |
| chr$4 | 92540 to 92890 | Rifin |
| chr$4 | 95830 to 96460 | Stevor |
| chr$4 | 133560 to 133840 | Surface asso. interspersed gene Surfin 4.1 |
| chr$4 | 133910 to 134190 | Surface asso. interspersd gene Surfin 4.1 |
| chr$4 | 162500 to 163400 | Rifin |
| chr$4 | 165690 to 166250 | Stevor |
| chr$4 | 171900 to 172400 | pfemP1 upstream |
| chr$4 | 172900 to 173500 | pfemP1 |
| chr$4 | 553140 to 553280 | pfemP1 |
| chr$4 | 553490 to 553560 | pfemP1 |
| chr$4 | 619530 to 619830 | pfemP1 upstream |
| chr$4 | 621000 to 621240 | pfemP1 downstream |
| chr$4 | 942550 to 942620 | pfemP1 |
| chr$4 | 942830 to 942900 | pfemP1 |
| chr$4 | 943180 to 943250 | pfemP1 |
| chr$4 | 943460 to 943530 | pfemP1 |
| chr$5 | 29900 to 30400 | Rifin |
| chr$5 | 545800 to 546120 | Rab1b GTPase |
| chr$5 | 773280 to 773600 | Intergenic region |
| chr$5 | 790400 to 790680 | Mito. ribosomal prot L14 |
| chr$5 | 857300 to 857700 | S-adenyl cystein lyase |
| chr$5 | 1301280 to 1301760 | Early transcribed Mb prot. 5 ETRAMP 5 |
| chr$5 | 1333400 to 1333520 | pfemP1 Truncated |
| chr$6 | 101800 to 102400 | Elongation factor G |
| chr$6 | 399800 to 399880 | Mito. cardiolipin synthase |
| chr$6 | 493110 to 493200 | potential conserved protein |
| chr$6 | 493700 to 493800 | potential conserved protein |
| chr$6 | 558440 to 558560 | Intergenic region |
| chr$6 | 623200 to 623500 | enoyl acyl carrier reductase |
| chr$6 | 731430 to 731970 | pfemP1 |
| chr$6 | 735840 to 736380 | Stevor |
| chr$6 | 738360 to 739080 | Stevor |
| chr$6 | 741510 to 741690 | Rifin |
| chr$6 | 741960 to 742230 | Rifin |
| chr$6 | 880290 to 880390 | Conserved Apicomplexan protein pfs77 |
| chr$6 | 880560 to 880700 | Conserved Apicomplexan protein pfs77 |
| chr$6 | 1333080 to 1333350 | Stevor |
| chr$6 | 1333620 to 1333890 | Stevor |
| chr$6 | 1336300 to 1336700 | Rifin |
| chr$6 | 1336800 to 1337200 | Rifin |
| chr$1 | 60600 to 61150 | Rifin |
| chr$1 | 478000 to 478500 | rrna encoding |
| chr$2 | 204000 to 204500 | 5'-3' exonuclease |
| chr$2 | 287800 to 288100 | Intergenic region |
| chr$3 | 232900 to 233100 | conserved protein |
| chr$3 | 366900 to 367500 | HSP-90 homolog_1 |
| chr$3 | 798900 to 799100 | Ubiquitin ligase |
| chr$3 | 854400 to 854800 | Intergenic region |
| chr$3 | 855000 to 855400 | Intergenic region |
| chr$4 | 95800 to 96400 | Stevor |
| chr$4 | 157000 to 157500 | Intergenic region |
| chr$4 | 166000 to 166600 | Stevor |
| chr$4 | 171880 to 172400 | Intergenic region |
| chr$4 | 620900 to 621300 | PfemP1 |
| chr$5 | 712700 to 712900 | 60S ribosomal protein L8 |
| chr$5 | 790390 to 790700 | Mito. ribosomal protein L14 |
| chr$6 | 204500 to 204700 | Glyoxalase 1 |
| chr$6 | 399790 to 399900 | Mito. cardiolipin synthase |
| chr$6 | 558300 to 558400 | Intergenic region |
| chr$6 | 558420 to 558600 | Intergenic region |
| chr$6 | 723000 to 723200 | PfemP1 |
| chr$6 | 912125 to 912650 | RNA methyl transferase |
| chr$6 | 1357200 to 1357700 | pfemp1 |
| chr$7 | 428100 to 428450 | DNA dep. RNA pol 2 |
| chr$7 | 574800 to 575150 | pfemp1 region |
| chr$7 | 653100 to 653400 | pfemp1 region |
| chr$7 | 742200 to 742500 | cal-cam kinase |
| chr$8 | 94100 to 94500 | non protein coding |
| chr$8 | 94900 to 95200 | non protein coding |
| chr$8 | 95200 to 95600 | Intergenic region |
| chr$8 | 95740 to 95980 | non protein coding |
| chr$8 | 320070 to 320300 | Intergenic region |
| chr$9 | 318220 to 318700 | Arginase |
| chr$9 | 440620 to 440980 | FHA domain protein |
| chr$9 | 880660 to 881100 | conserved protein unknown function |
| chr$9 | 1459320 to 1459780 | PHIST domain protein |
| chr$10 | 60700 to 61250 | pfemp1 truncated |
| chr$10 | 261850 to 262150 | Intergenic region |
| chr$10 | 266190 to 266330 | conserved protein unknown function |
| chr$10 | 266350 to 266550 | conserved protein unknown function |
| chr$10 | 1487270 to 1487500 | Intergenic region |
| chr$11 | 131570 to 131800 | Early transcribed mb protein 11.2 |
| chr$11 | 584250 to 584500 | Falcipain-3 |
| chr$12 | 57110 to 57350 | pfemp1 |
| chr$12 | 764210 to 764380 | close to pfemp1 |
| chr$12 | 1691180 to 1691600 | close to pfemp1 |
| chr$12 | 1692610 to 1692850 | close to pfemp1 |
| chr$12 | 1817360 to 1817600 | Intergenic region |
| chr$12 | 1983600 to 1983980 | PF drosophila nmda1 protein |
| chr$12 | 2190930 to 2191500 | Rifin |
| chr$13 | 525310 to 525540 | Intergenic region |
| chr$13 | 911830 to 912100 | conserved protein unknown function |
| chr$13 | 1044520 to 1044670 | Intergenic region |
| chr$13 | 1044740 to 1044850 | PRPP synthase |
| chr$13 | 1309750 to 1310130 | Signal peptidase |
| chr$13 | 1415230 to 1415550 | MSP7 -like |
| chr$13 | 2512810 to 2512960 | MAL13P1 |
| chr$13 | 2659710 to 2659820 | SRP receptor alpha SU |
| chr$13 | 2833750 to 2833850 | Rifin |
| chr$13 | 2834230 to 2834420 | Rifin-Stevor |
| chr$14 | 39930 to 40210 | Intergenic region |
| chr$14 | 46990 to 47500 | PEXEL |
| chr$14 | 373010 to 373190 | PF14_0093_rev |
| chr$14 | 516070 to 516230 | Intergenic region |
| chr$14 | 516550 to 516650 | N-myristoyl tfase |
| chr$14 | 732000 to 732650 | conserved protein unknown function |
| chr$14 | 739350 to 739700 | conserved protein unknown function |
| chr$14 | 958250 to 958410 | Intergenic region |
| chr$14 | 1094240 to 1094500 | Intergenic region |
| chr$14 | 1673290 to 1673410 | PF14_0390_rev |
| chr$14 | 1681550 to 1681790 | ser/thr kinase |
| chr$14 | 2014590 to 2014790 | conserved protein unknown function |
| chr$14 | 2553570 to 2553810 | cytochrome C1 precursor |
| chr$14 | 2657350 to 2657590 | PF14_0624_rev |
| chr$14 | 2768330 to 2768570 | Intergenic region |
| chr$14 | 2899720 to 2899910 | Intergenic region |
| chr$14 | 3221030 to 3221510 | PF14_0755_changed |
| chr$14 | 3272650 to 3272910 | Rifin |
